# Quantifying cortical development in typically developing toddlers and young children, 1–6 years of age

**DOI:** 10.1016/j.neuroimage.2017.04.010

**Published:** 2017-06

**Authors:** Justin Remer, Elise Croteau-Chonka, Douglas C. Dean, Sara D’Arpino, Holly Dirks, Dannielle Whiley, Sean C.L. Deoni

**Affiliations:** Advanced Baby Imaging Lab, School of Engineering, Brown University, Providence, RI 02912, United States

## Abstract

Cortical maturation, including age-related changes in thickness, volume, surface area, and folding (gyrification), play a central role in developing brain function and plasticity. Further, abnormal cortical maturation is a suspected substrate in various behavioral, intellectual, and psychiatric disorders. However, in order to characterize the altered development associated with these disorders, appreciation of the normative patterns of cortical development in neurotypical children between 1 and 6 years of age, a period of peak brain development during which many behavioral and developmental disorders emerge, is necessary. To this end, we examined measures of cortical thickness, surface area, mean curvature, and gray matter volume across 34 bilateral regions in a cohort of 140 healthy children devoid of major risk factors for abnormal development. From these data, we observed linear, logarithmic, and quadratic patterns of change with age depending on brain region. Cortical thinning, ranging from 10% to 20%, was observed throughout most of the brain, with the exception of posterior brain structures, which showed initial cortical thinning from 1 to 5 years, followed by thickening. Cortical surface area expansion ranged from 20% to 108%, and cortical curvature varied by 1–20% across the investigated age range. Right-left hemisphere asymmetry was observed across development for each of the 4 cortical measures. Our results present new insight into the normative patterns of cortical development across an important but under studied developmental window, and provide a valuable reference to which trajectories observed in neurodevelopmental disorders may be compared.

## Highlights

• Analysis of cortical thickness, surface area, curvature, and gray matter volume.
• Region specific trajectories of cortical maturation in infants and children.
• Analysis of significant asymmetry during early brain development.
• Differential brain development based on sex.

## Introduction

Human cortical development is a complex process comprising both gross morphometric change and microstructural progressions. Throughout late gestation, rapid synaptogenesis results in an overabundance of synapses (up to 150% of adult values) that are subsequently pruned throughout childhood and adolescence (Huttenlocher, 1979, Huttenlocher, 1990). Gyrification of the cortex also begins early in development (by 10 weeks gestation) and continues throughout *in utero* development and postnatally following birth (White et al., 2010). Advancement of the cortical myeloarchitecture also contributes to the changing microstructure and morphology, and is associated with developing brain function and neuroplasticity (Brody et al., 1987, Leipsic, 1901, Yakovlev and Lecours, 1967). These morphometric and microstructural changes can be observed on MRI, and can be indirectly quantified by metrics including cortical thickness, surface area, volume, and curvature (Croteau-Chonka et al., 2016, Deoni et al., 2015, Grydeland et al., 2013).

In neonates and infants, studies of cortical development have focused on changing grey matter volume (Gilmore et al., 2012, Knickmeyer et al., 2008), gyrification (Li et al., 2014), deep sulcal landmark maturation (Meng et al., 2014), thickness and surface area maturation (Lyall et al., 2015), as well as folding and fiber density (Nie et al., 2014). In general, studies suggest a complex pattern of development that varies based on anatomical location and cortical metric. In addition, across early development, cortical maturation exhibits regionally-specific asymmetry between the left and right hemispheres (Li et al., 2014, Nie et al., 2014). These changes continue throughout childhood and adolescence, with cortical thickness following different trajectories of thinning depending on region, cortex type (Shaw et al., 2008), and gender (Sowell et al., 2004).

Prior studies in children, adolescents and adults have consistently linked differences in cortical structure, morphology, and morphometry to differences in measures of cognitive and behavioral performance. For example, Shaw and colleagues showed differential rates of thinning across childhood and adolescence in individuals with high vs low general cognitive ability (i.e., IQ) (Shaw et al., 2006); in association with attention and other executive functions (Blakemore and Choudhury, 2006, Shaw et al., 2011); and in memory decline in normal aging and dementia (Dickerson et al., 2009). In studies of learning and plasticity, cortical changes are also consistently observed in association with training (Boyke et al., 2008, Colom et al., 2016, Draganski et al., 2004, Draganski and May, 2008).

Given the importance of cortical structure and development on brain function, it is not surprising that abnormalities in cortical maturation have been implicated in developmental, intellectual, psychiatric, and neurologic disorders. For example, cross-sectional and longitudinal differences in cortical thickness, mean curvature, surface area, and/or volume have been shown in schizophrenia (Rapoport et al., 2012), autism (Courchesne et al., 2007, Hazlett et al., 2012), and attention deficit hyperactivity disorder (Shaw et al., 2011, Shaw et al., 2012), amongst others. Many of these disorders are believed to originate during early neurodevelopment, and likely are associated with, or result from, abnormal brain and cortical maturation.

Despite the number and variety of prior imaging studies, there remains limited information on how the cortex matures throughout early childhood, specifically between the ages of 1 and 6 years (see Supplementary table 1 for a list of studies on the anatomical development of cortex in other age ranges as well as Walhovd et al. (2017) for a list of recent studies on cortical thickness development) (Giedd et al., 1999, 2015; Gogtay et al., 2004; Hill et al., 2010a, Hill et al., 2010b; Lenroot et al., 2007; Raznahan et al., 2014; Shi et al., 2011; Storsve et al., 2014; Tamnes et al., 2017; Walhovd et al., 2017). This period of development is important as it coincides with the emergence and initial refinement of cognitive and behavioral skills and abilities, and is also when many developmental behavioral and intellectual disorders are believed to first manifest.

Here, we aimed to address this gap and quantify metrics of cortical maturation, including thickness, surface area, volume, and curvature, in a large cohort (n=140, 62 female) of healthy and neurotypical children between 1 and 6 years of age. Combining high-resolution magnetic resonance imaging (MRI) data with Freesurfer reconstruction, we analyzed 34 cortical regions per hemisphere and fit cortical growth patterns using linear and nonlinear models. Parameter estimates from the fitting enabled reconstruction of cortical development trajectories and derivation of growth rate patterns. Data presented here provide new insight into childhood cortical development, and lay the foundation for future studies on longitudinal and atypical cortical development.

## Methods

140 healthy and typically-developing children between 350 and 2180 days (approximately 1 to 6 years), corrected to a 40 week gestation, were recruited from the local community as part of a larger longitudinal study of healthy neurodevelopment (Deoni et al., 2012). To focus on healthy development, children with known risk factors for abnormal development were excluded at time of recruitment. Specific inclusion criteria included: uncomplicated healthy singleton birth between 37 and 42 weeks gestation; no abnormalities on fetal ultrasound; no complications during pregnancy; no illicit drug or alcohol use during pregnancy; no admissions to the neonatal intensive care unit; no reported history of neurological events or disorders (e.g. head trauma, epilepsy, etc.); and no familial record of psychiatric or neurological disorders. Criteria were confirmed at time of enrollment through parental interviews. Medical and family history questionnaires were completed to obtain additional demographic and medical information on participating children (Table 1). The host institution's internal review board approved this study and informed consent was obtained from each participating family. Additional inclusion criteria consisted of MRI images with no evidence of motion artifact or ghosting on visual inspection.

**Table 1:** Participant demographic information.

Gender	Male (n)	78
	Female (n)	62
Racial background	Caucasian (n)	92
	African American (n)	11
	Asian (n)	2
	Mixed race (n)	20
Ethnic background	Hispanic (n)	30
	Non- Hispanic (n)	98
Mean maternal SES^a^	5.8±1.1	
Marital status at scan	Married (n)	91
	Single (n)	23
	Living together (n)	10
	Separated (n)	7
	Divorced (n)	2
Mean scan age (days)	1010.7±528.2	
Birth type	Vaginal (n)	82
	Cesarean (n)	41
Mean gestation age (days)	274.8±9.5	
Mean birth height (inches)	20.2±1.5	
Mean birth weight (ounces)	117.9±17.5	
Feeding method	Total Nursed (n)	52
	Total Bottle Fed (n)	27
	Total Both (n)	44
Mean maternal age at delivery (days)	10,885.2 ± 2360.7	
Average mullen composite score	ELC	101
	NVDQ	104
	VDQ	101

1.1 – Less than 7th grade.

1.2 – Junior high school.

1.3 – Partial high school.

1.4 – High school graduate.

1.5 – Partial college or standardized training.

1.6 – College graduate.

1.7 – Graduate training (Masters, Ph.D.).

a SES Determination:

### Image acquisition

All MRI data were acquired at a single site on the same 3T Siemens Tim Trio MRI scanner with a 12-channel head RF coil array. High-resolution structural MRI images were obtained for children either during natural and non-sedated sleep or whilst watching a favorite movie (Dean et al., 2014). In order to minimize acoustic noise levels and ensure children remained asleep for the scan, gradient slew rates and amplitudes were decreased to approximately 30% and 75% of their maximal values, respectively. Additional noise reduction measures included the use of electrodynamic headphones (MR Confon, Germany) and a removable sound-insulating foam insert (Ultra Barrier HD Composite, UltraBarrier USA) placed inside the scanner bore. Children were also swaddled with an appropriately sized MedVac vacuum immobilization bag (CFI Medical Solutions, USA) to help minimize subtle body movement.

The structural MRI protocol involved acquisition of inversion prepared spoiled gradient echo (IR-SPGR) images at a 5° flip angle, 16 ms repetition time (TR), 6.9 ms echo time (TE), 950 ms inversion time (TI), and a voxel resolution of 1.2×1.2×1.2 mm^3. The acquisition matrix and field of view were varied according to head size to maintain the same voxel resolution across all ages (Deoni et al., 2012, Deoni et al., 2015). A single MRI per subject was used in this study.

### Quality control

As detailed in Backhausen et al. (2016) quality control of structural MRI plays a significant role in assessing results from neurodevelopmental studies. For our cohort, image quality control were assessed in the following ways. First, MRI images were visually inspected for motion artifacts, including degradation of image sharpness, ringing, or poor contrast to noise ratio. Participants with images that failed this initial visual inspection then either underwent repeat data acquisition or were rescheduled for a repeat MRI at a later time. Second, prior to Freesurfer cortical reconstruction, all acquired MRI data sets were again examined by 3 independent reviewers (JR, EC, SD), looking for evidence of motion artifact as well as poor image quality with the criteria stated above. If image inspection failed from either reviewer that participant was not included in the cohort for this study.

### Cognitive assessments

To evaluate general cognitive ability, all children were assessed using the Mullen Scales of Early Learning (MSEL; Mullen, 1995) within one week of the MRI scan. The MSEL is a standardized and population-normed tool for assessing visual, motor, and verbal abilities in children from birth to 68 months of age. These measures can be combined into an overall early learning composite (ELC), which reflects general cognitive ability, as well as verbal and non-verbal development quotients (VDQ and NVDQ, respectively) reflecting language and motor skills. For this study, our cohort of children had an average ELC score of 101, NVDQ of 104, and a VDQ of 101 consistent with typical development (Table 1). In addition, validated screening tools, including the modified checklist for Autism in Toddlers (M-CHAT) and the communication and Symbolic Behavior Scales Developmental Profile (CSBS-DP), were used to identify and exclude children with clinically concerning behaviors. No participant was excluded on these bases.

### Cortical reconstruction

Standard image pre-processing included correction of low frequency signal intensity variation (RF coil bias field) using the Advanced Normalization Tools (ANTs) nonparametric non-uniform normalization (N3) tool (Sled et al., 1998). Cortical thickness, surface area, mean curvature, and volume were measured for 34 bilateral regions using the surface mesh based cortical modeling software package, Freesurfer (Dale et al., 1999; Fischl et al., 1999). At each step in the processing protocol, images were visually inspected and, when required, manually edited using gcut (http://freesurfer.net/fswiki/FsTutorial/SkullStripFix_freeview), which corrects any improper skull stripping and removes non-brain tissue including dura and eye signal. Additional details on the Freesurfer cortical reconstruction protocol have been described previously (Dale et al., 1999, Fischl et al., 1999). To ensure accurate cortical segmentation we used a custom template that our group has previously developed for Freesurfer segmentation of the age range of the current sample (Croteau-Chonka et al., 2016; Deoni et al., 2015) The custom template was generated using Freesurfer's make_average_subject command (http://surfer.nmr.mgh.harvard.edu/fswiki/make_average_subject) and included 5 age-matched males and 5 age-matched females from each age-range (for a total of 50 participants). This was done to avoid biasing the template to any particular gender or age range.

### Trajectories of cortical development

In order to characterize cortical maturation, we constructed developmental trajectories of cortical thickness; surface area; curvature; and volume using the outputs from the Freesurfer cortical reconstruction protocol. For each investigated region, mean cortical metrics were plotted against each child's gestation corrected age, while linear, quadratic, and logarithmic models were fit to the data. Model fits were compared using the Bayesian Information Criterion (BIC) (Schwarz, 1978) to determine the most parsimonious fit. Cubic, higher order polynomials and spline based models were not included in this analysis due to our group's preliminary analysis of cortical thickness maturation (Croteau-Chonka et al., 2016, Deoni et al., 2015) demonstrating that cortical thickness predominantly follows logarithmic trajectories. Model formulas consisted of:

(1) $$\mathrm{Cortical}\;\mathrm{Parameter}=\beta_{\mathrm{Ln}\;1}*\;\ln(\mathrm{participant}\;\mathrm{age})+\beta_{\mathrm{Ln}\;2}$$

(2) $$\mathrm{Cortical}\;\mathrm{Parameter}=\beta_{Q1}*{\left(\mathrm{participant}\;\mathrm{age}\right)}^{2}+\beta_{Q2}*\left(\mathrm{participant}\;\mathrm{age}\right)+\beta_{Q3}$$

(3) $$\mathrm{Cortical}\;\mathrm{Parameter}=\beta_{L1}*\left(\mathrm{participant}\;\mathrm{age}\right)+\beta_{L2}$$

where cortical parameter is either cortical thickness, cortical surface area, cortical mean curvature, or cortical gray matter volume, and participant age is in days corrected to a 40 week gestation. Percent increase of cortical maturation was calculated for each region by comparing the population cortical value at 360 and 2180 days using the curve of best fit.

### Sex differences in cortical maturation

In order to consider possible differences in development based on sex our cohort was divided between male and female participants and cortical thickness, surface area, mean curvature and volume were plotted as a function of participant age for both groups. Trajectories of cortical development were generated for each group using region specific growth curves based on the models of best fit and F-tests were used to determine significance of modeling cortical development categorized by sex. Significance was defined for each cortical metric as α<0.000735 (p<0.05 corrected for 34 brain regions per hemisphere).

### Asymmetry in cortical maturation

To characterize asymmetries in cortical maturation, an Asymmetry Index ( AI=[left–right]/[left+right]) was calculated for each examined measure (cortical thickness, surface area, curvature, and volume) in all 34 brain regions and for each individual. AI values were then plotted against each individual's gestation corrected age. A single sample t-test was performed to identify brain regions exhibiting significant asymmetry. Significance was defined as α<0.0014 (p<0.05 Bonferroni corrected for 34 brain regions). Further asymmetry analysis focused on identifying specific age intervals of significant asymmetric cortical development. AI values of all four measures of cortical population were analyzed with a sliding window approach. Ordering subjects by age, the first 50 children (#1–50) were grouped and the mean AI calculated; then subjects #2–51 were grouped and the mean AI calculated, and so forth. Significant windows of asymmetry were determined through a single sample t-test and significance was defined as α<1.6*10^−5^ (p<0.05 corrected for 34 brain regions each with 50 subjects per window).

## Results

### Cortical maturation trajectories

To characterize changes in cortical thickness, surface area, curvature and volume from 1 to 6 years, linear, quadratic, and logarithmic models were fit to the data. Model residuals were compared using the Bayesian information Criteria (BIC) to identify the model of best fit. Results of this analysis (Supplementary Table 2–5) show region-specific developmental variations in cortical thickness, surface area, curvature and volume.

**Fig. 1:**
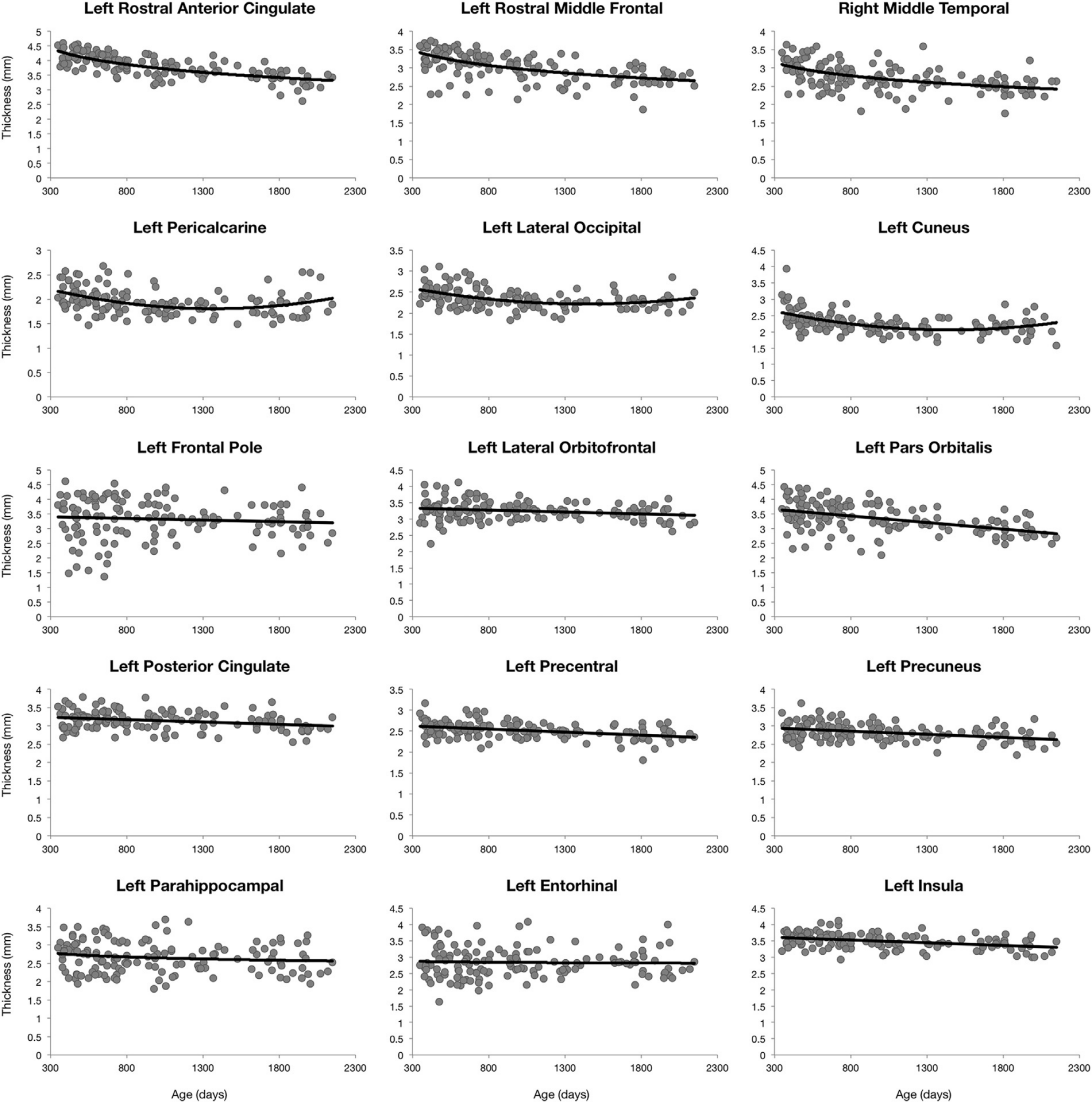
Region specific cortical thickness trajectories for a selection of anatomical regions. Points correspond to subject specific thickness value and the line corresponds to the trend line of best fit from BIC analysis.

Cortical thickness growth curves predominantly follow logarithmic trajectories (see Fig. 1) revealing diffuse slow bilateral cortical thinning. Exceptions include posterior brain regions (pericalcarine cortex, lateral occipital cortex, and the cuneus), which follow quadratic trajectories; and frontal and parietal regions (including frontal pole, lateral orbitofrontal, parsorbitalis, posterior cingulate, precentral gyrus, and precuneus), which follow a linear trend.

Decreases in cortical thickness ranging from 10% to 20% were observed in most brain regions with exceptions in posterior brain regions which revealed 50–80% increases. (Supplementary Table 2).

**Fig. 2:**
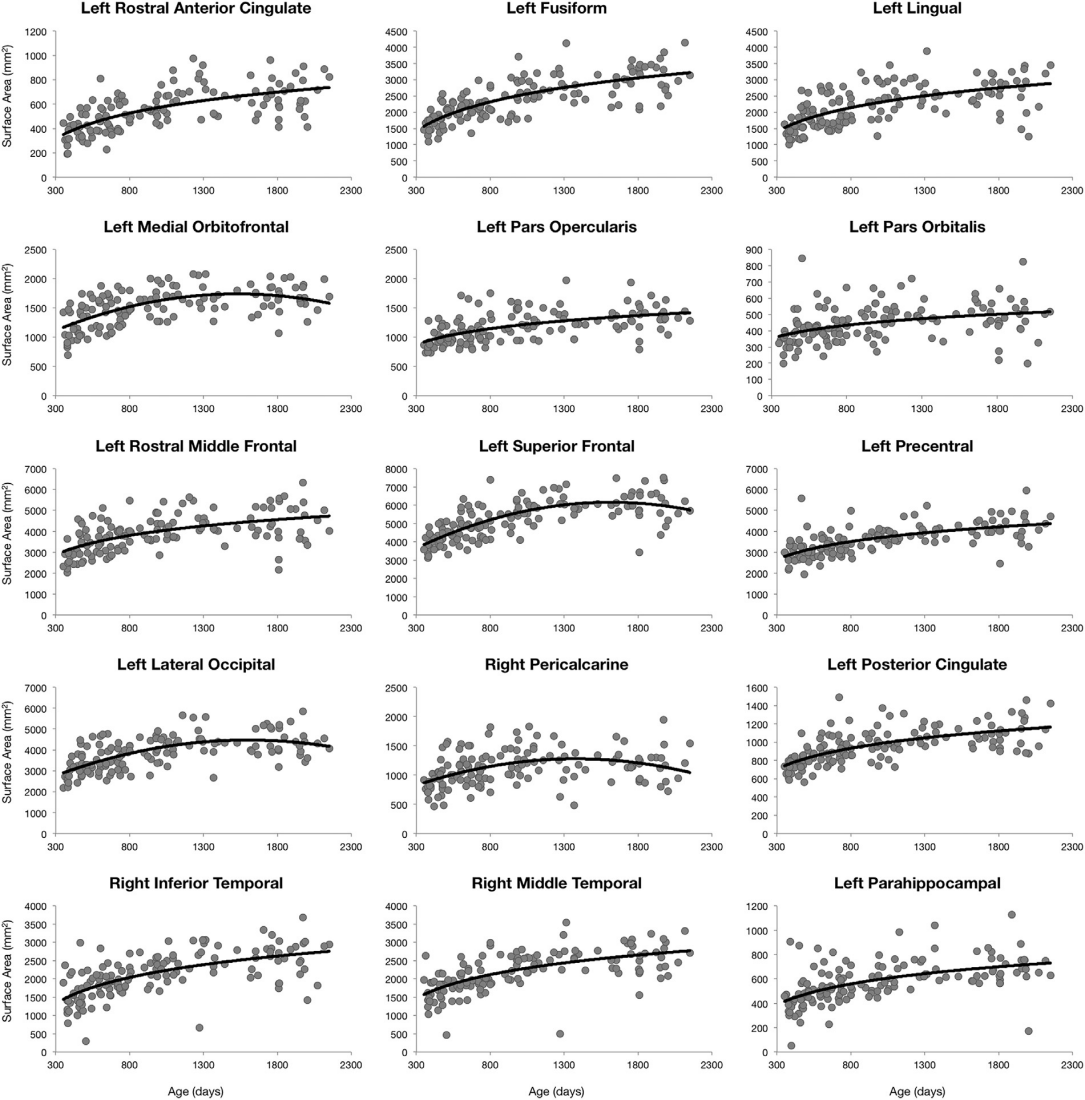
Region specific cortical surface area trajectories for a selection of anatomical regions. Points correspond to subject specific surface area value and the line corresponds to the trend line of best fit from BIC analysis.

We observed that cortical surface area generally followed a logarithmic trajectory, with bilateral expansion in all neuroanatomical locations (see Fig. 2). However, quadratic trajectories were also observed in right frontal structures (medialorbitofrontal cortex, parsopercularis, parsorbitalis, rostral middle frontal gyrus, superior frontal gyrus, and precentral gyrus), posterior structures (bilateral lateraloccipital cortex and right pericalcarine), and medial structures (posterior cingulate, rostral middle cingulate, and isthmus of the cingulate). Cortical surface area expansion between 1 and 6 years of age ranged from 20% to 108% (Supplementary Table 3) with the greatest increases observed in bilateral rostral anterior cingulate (108%), left fusiform gyrus (102%), and left lingual gyrus (87%).

**Fig. 3:**
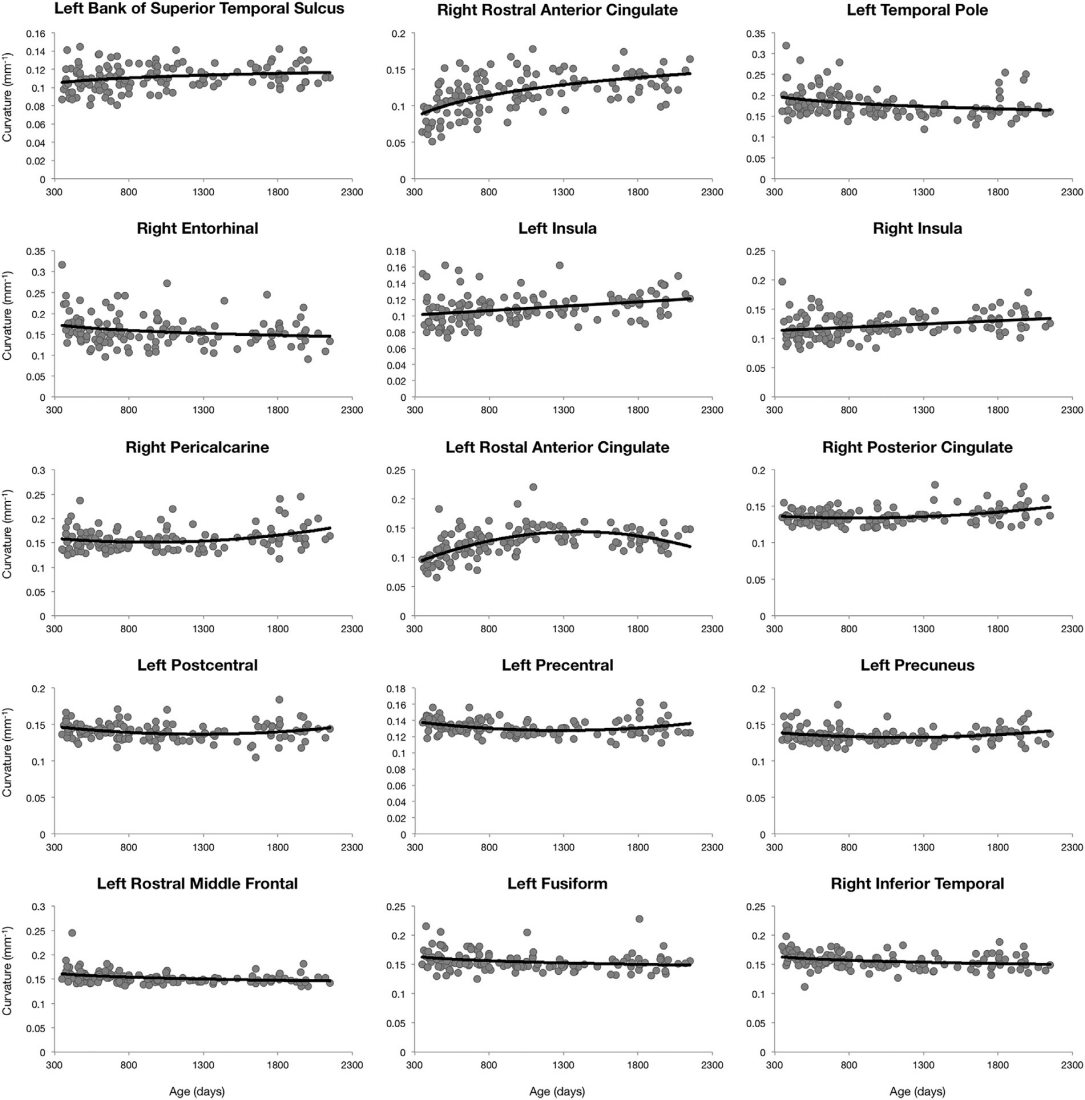
Region specific cortical mean curvature trajectories for a selection of anatomical regions. Points correspond to subject specific mean curvature value and the line corresponds to the trend line of best fit from BIC analysis.

Maturation in cortical curvature followed a combination of linear, quadratic, and logarithmic trajectories, but predominantly followed logarithmic trajectories (Fig. 3). Changes in curvature were more modest with respect to those seen for thickness and surface area, with an average decrease of approximately 1% across all regions (Supplementary Table 4). Exceptions include the left bank of the superior temporal sulcus (10% increase), the right rostral anterior cingulate (61% increase), the left temporal pole (16% decrease), and right entorhinal cortex (15% decrease), which all followed logarithmic growth trajectories. Quadratic trajectories with initial decreasing but later increasing curvature were observed in the right pericalcarine cortex (15%) and left rostral anterior cingulate (21%), and right posterior cingulate (10%). The majority of anatomical locations that followed linear growth curves exhibited increasing curvature with the greatest increase observed in the insula (19% and 18% in left and right respectively).

**Fig. 4:**
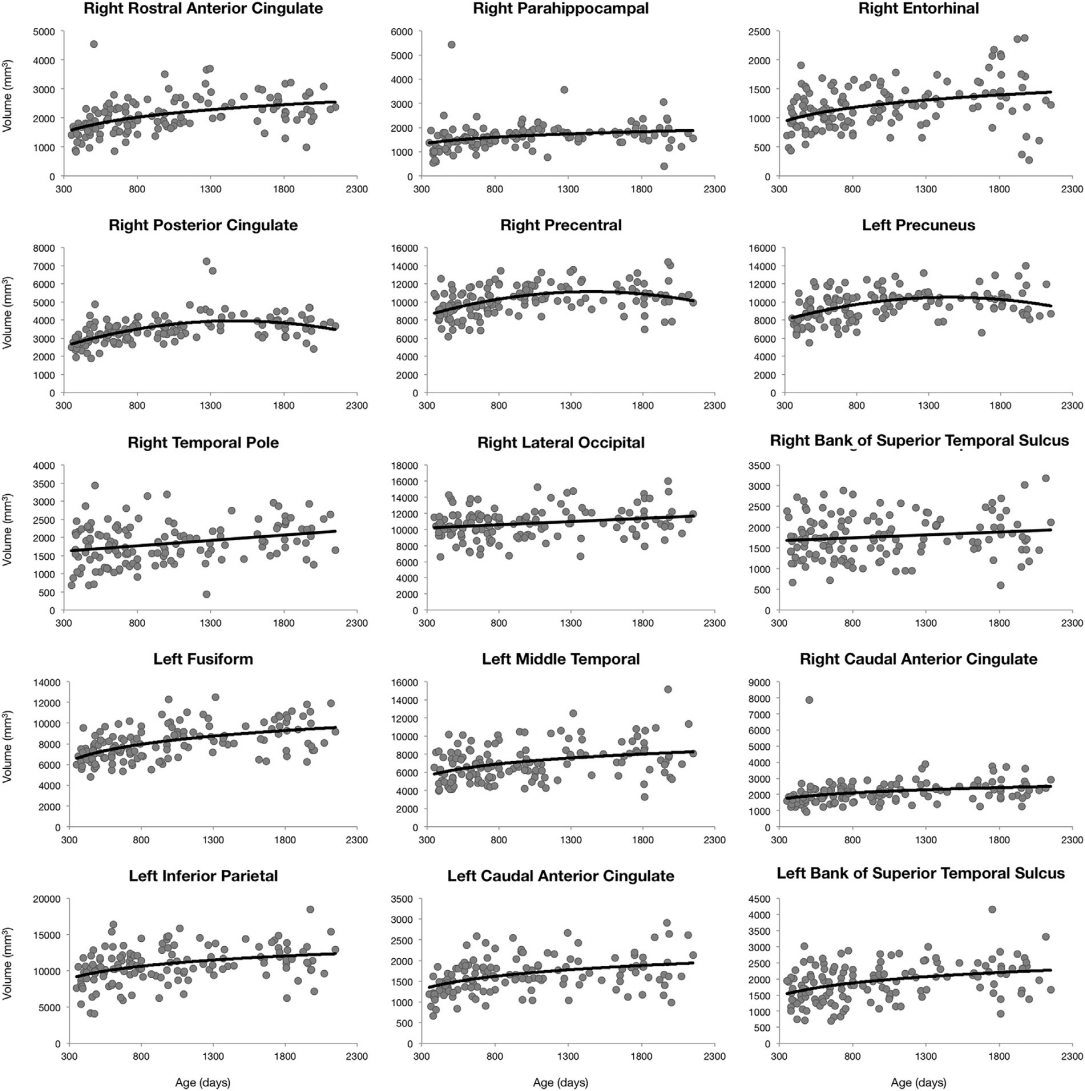
Region specific cortical gray matter volume trajectories for a selection of anatomical regions. Points correspond to subject specific gray matter volume value and the line corresponds to the trend line of best fit from BIC analysis.

Unsurprisingly, cortical volume was found to increase throughout the cortex, following predominantly logarithmic trajectories. An average increase of 20% was observed, with structures such as right rostral anterior cingulate (60%), bilateral parahippocampal gyrus (37% and 39% for right and left respectively) and right entorhinal cortex (50%) exhibiting larger increases over the studied age range (Supplementary Table 5). Quadratic growth curves were observed primarily in frontal and cingulate brain regions with peak volume values obtained around 5–6 years of age. Linear trajectories were observed in bilateral temporal poles revealing a constant rate in volume expansion across early childhood (Fig. 4).

### Cortical asymmetry

**Fig. 5:**
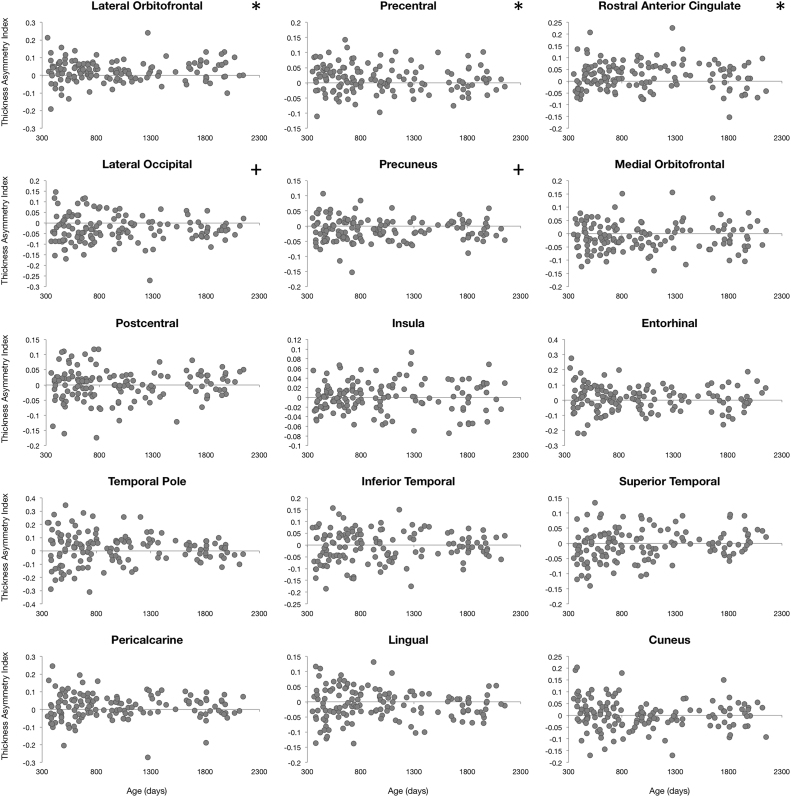
Analysis of cortical thickness asymmetry. Points correspond to asymmetry index calculated for each subject. * corresponds to regions with significant left lateralized asymmetry and + corresponds to regions with significant right lateralized asymmetry.

**Fig. 6:**
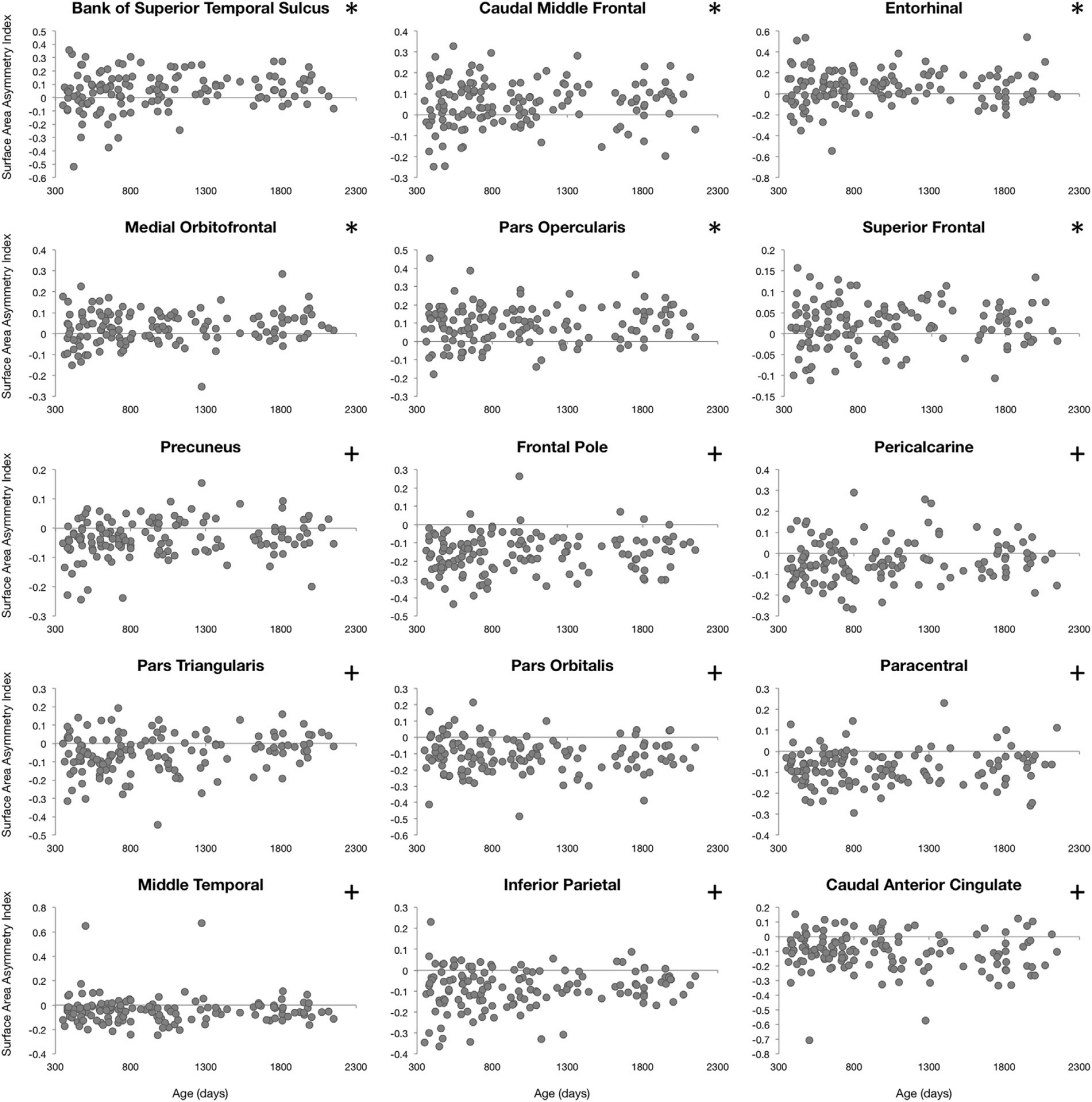
Analysis of cortical surface area asymmetry. Points correspond to asymmetry index calculated for each subject. * corresponds to regions with significant left lateralized asymmetry and + corresponds to regions with significant right lateralized asymmetry.

**Fig. 7:**
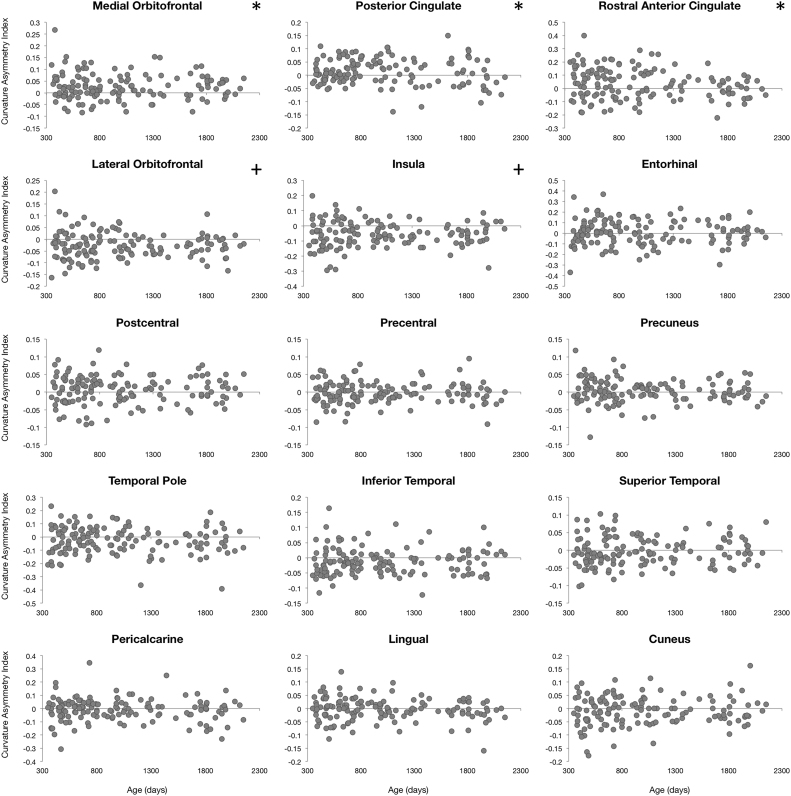
Analysis of cortical mean curvature asymmetry. Points correspond to asymmetry index calculated for each subject. * corresponds to regions with significant left lateralized asymmetry and + corresponds to regions with significant right lateralized asymmetry.

**Fig. 8:**
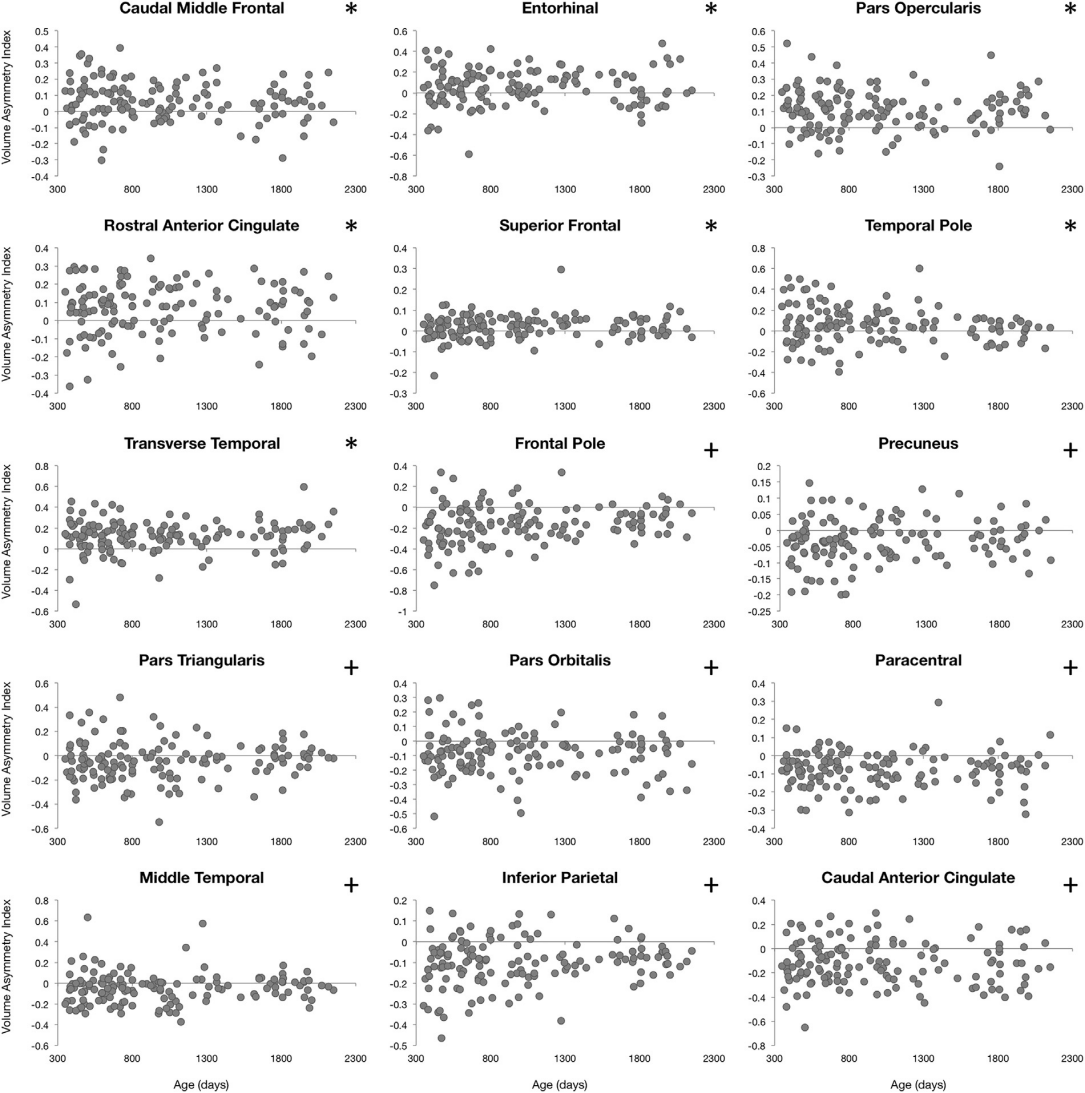
Analysis of cortical gray matter volume asymmetry. Points correspond to asymmetry index calculated for each subject. * corresponds to regions with significant left lateralized asymmetry and + corresponds to regions with significant right lateralized asymmetry.

Left-right hemisphere differences in cortical measurements were also observed (Fig. 5, Fig. 6, Fig. 7, Fig. 8). Significant left lateralized thickness was observed in the lateral orbitofrontal cortex, precentral gyrus, and rostral anterior cingulate; while significant right lateralized thickness was observed in the lateral occipital cortex and precuneus. With regards to surface area, significant left lateralized asymmetry was evident in the bank of the superior temporal sulcus, caudal middle frontal gyrus, entorhinal cortex, medial orbitofrontal cortex, parsopercularis, superior frontal gyrus, and transverse temporal gyrus. Significant right lateralized asymmetry was evident in the precuneus, frontalpole, pericalcarine cortex, parstriangularis, parsorbitalis, paracentral gyrus, middle temporal gyrus, inferior parietal cortex, and caudal anterior cingulate. Significant asymmetries in curvature were left lateralized in the medial orbitofrontal cortex, posterior cingulate, and rostral anterior cingulate; and significant asymmetries were right lateralized in the lateral orbitofrontal cortex and insula. Cortical gray matter volume asymmetries were significantly left lateralized in the caudal middle frontal gyrus, the entorhinal cortex, parsopercularis, rostral anterior cingulate, superior frontal gyrus, temporal pole, and transverse temporal. Right lateralized asymmetry of volume was observed in the frontal pole, precuneus, parstriangularis, parsorbitalis, paracentral gyrus, middle temporal gyrus, inferior parietal cortex, and caudal anterior cingulate.

**Fig. 9:**
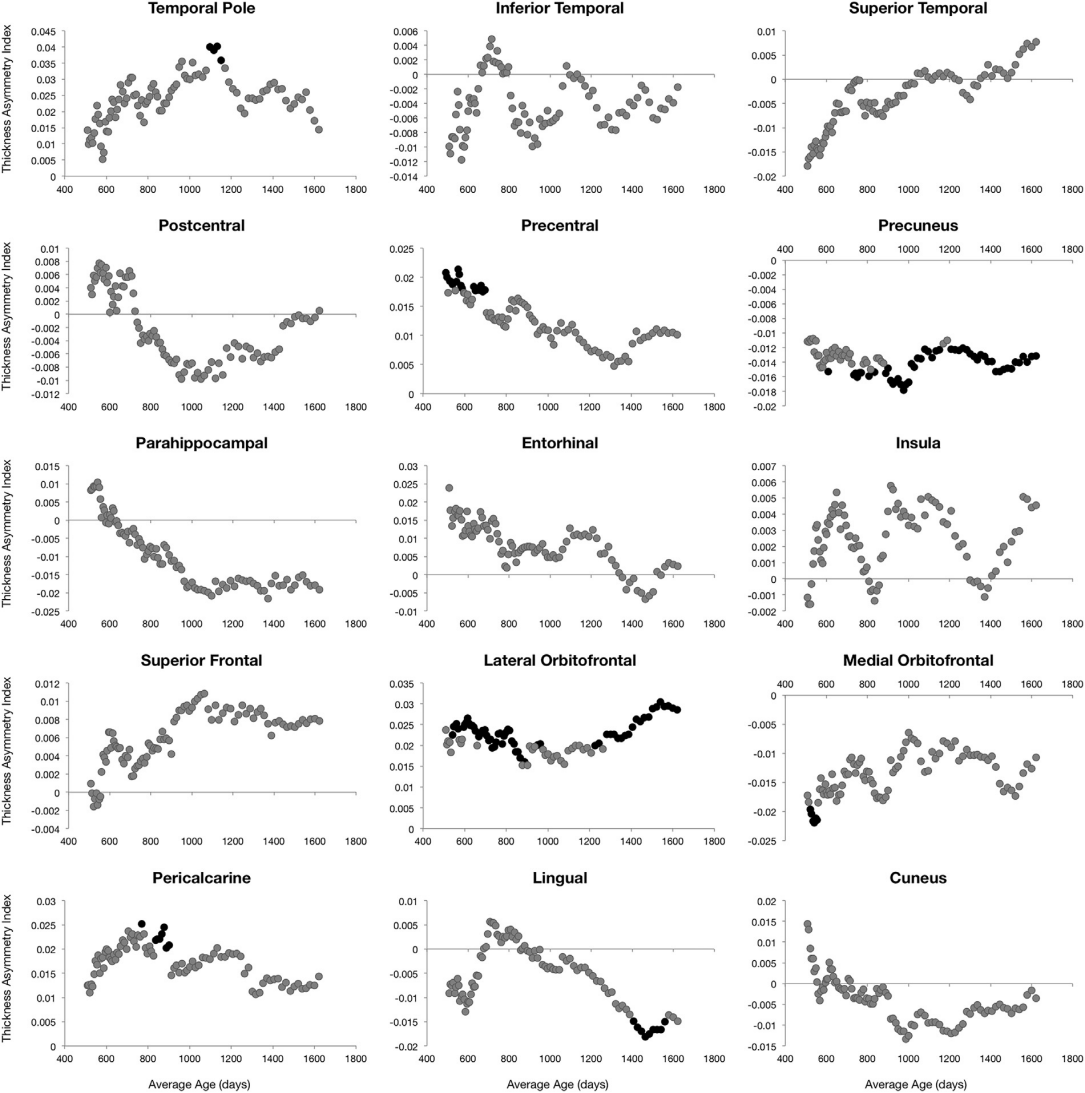
Sliding window analysis of cortical thickness asymmetry. The asymmetry index was calculated for a sliding and overlapping window of 50 children plotted with respect to mean age for the window. A single sample t-test was used to determine if window asymmetry index differed significantly from zero. Significant areas were defined by p<0.05 corrected for 34 brain regions each with 50 subjects per window and denoted with dark shading.

**Fig. 10:**
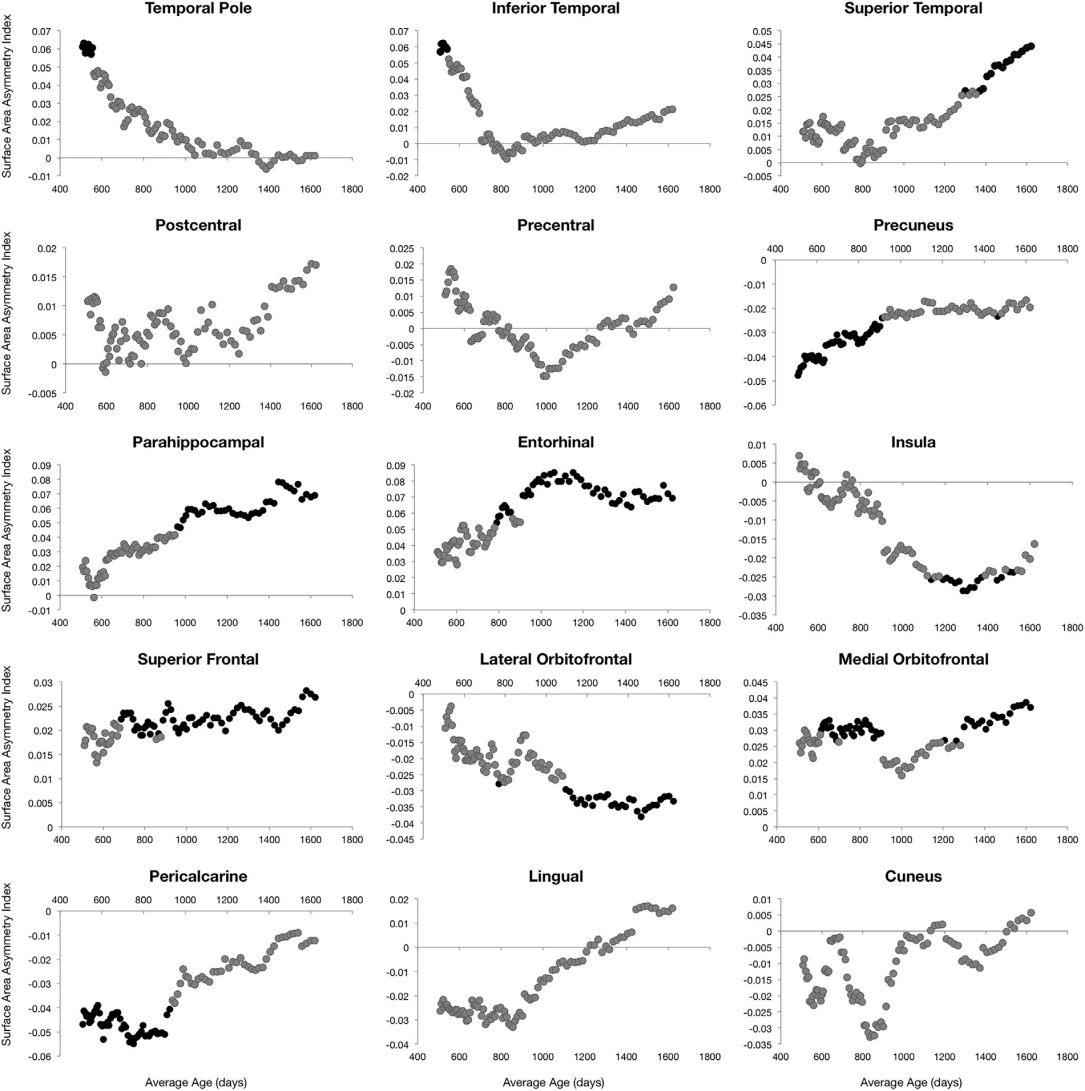
Sliding window analysis of cortical surface area asymmetry. The asymmetry index was calculated for a sliding and overlapping window of 50 children plotted with respect to mean age for the window. A single sample t-test was used to determine if window asymmetry index differed significantly from zero. Significant areas were defined by p<0.05 corrected for 34 brain regions each with 50 subjects per window and denoted with dark shading.

**Fig. 11:**
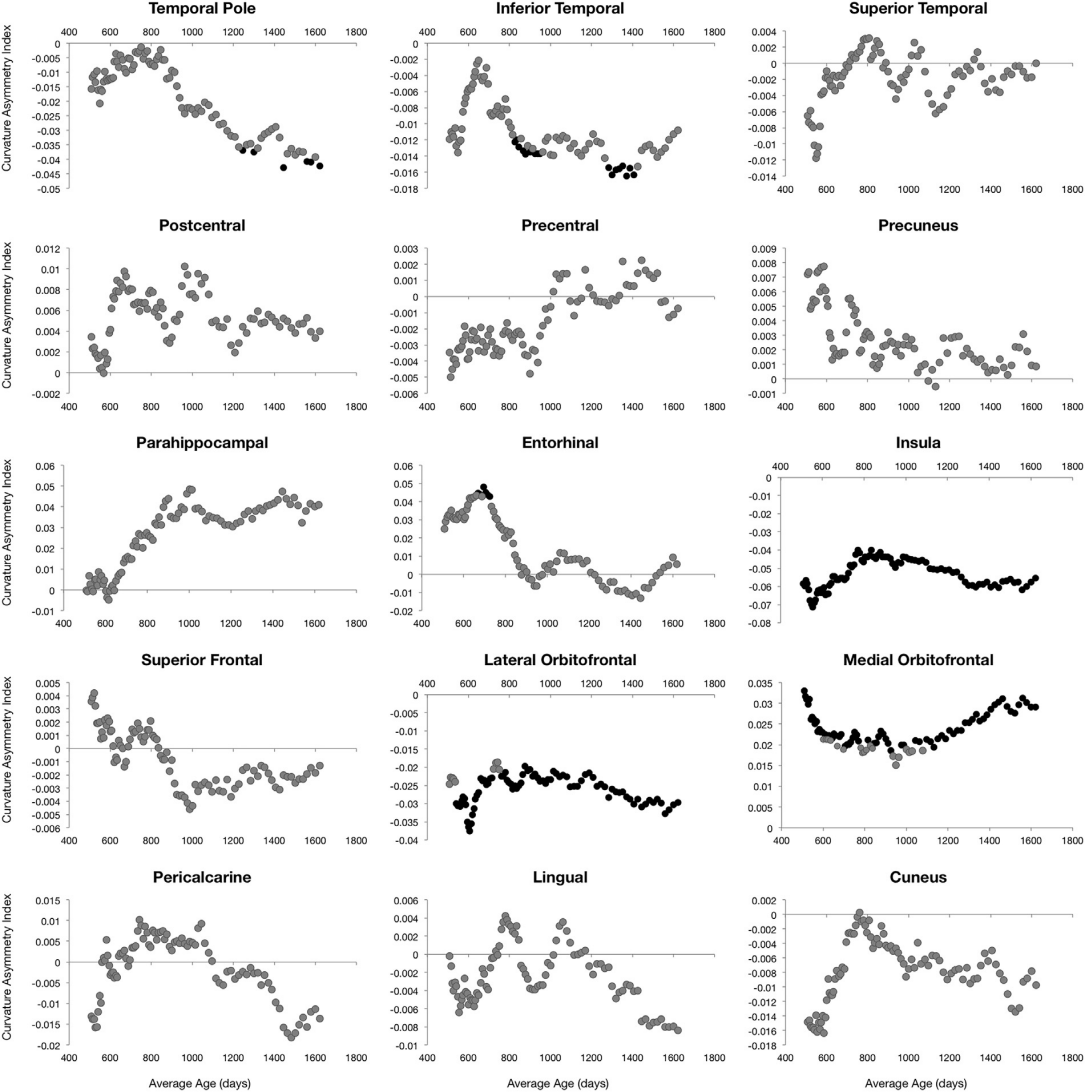
Sliding window analysis of cortical mean curvature asymmetry. The asymmetry index was calculated for a sliding and overlapping window of 50 children plotted with respect to mean age for the window. A single sample t-test was used to determine if window asymmetry index differed significantly from zero. Significant areas were defined by p<0.05 corrected for 34 brain regions each with 50 subjects per window and denoted with dark shading.

**Fig. 12:**
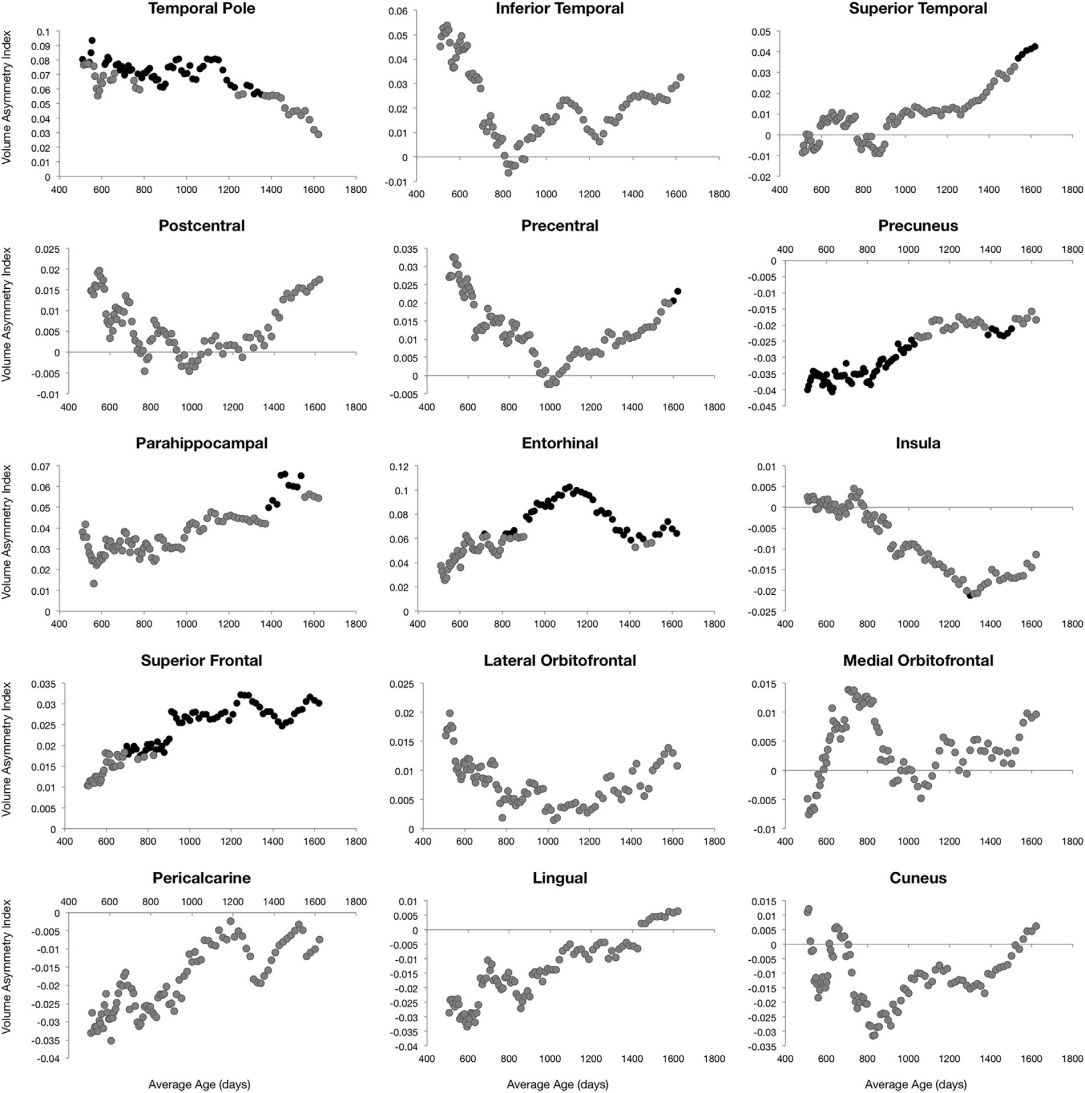
Sliding window analysis of cortical gray matter volume asymmetry. The asymmetry index was calculated for a sliding and overlapping window of 50 children plotted with respect to mean age for the window. A single sample t-test was used to determine if window asymmetry index differed significantly from zero. Significant areas were defined by p<0.05 corrected for 34 brain regions each with 50 subjects per window and denoted with dark shading.

For cortical thickness, sliding window analysis revealed significant early age asymmetries in the precentral gyrus and medial orbitofrontal cortex and significant late age asymmetries in the precuneus and lateral orbitofrontal cortex (Fig. 9). Age related asymmetries were observed in surface area development, with significant early age asymmetries in the temporal pole, inferior temporal gyrus, precuneus, and pericalcarine cortex, and significant late age related asymmetries were observed in the lateral and medial orbitofrontal cortex, entorhinal cortex, superior frontal gyrus, insula, and superior temporal gyrus (Fig. 10). For curvature, significant age related asymmetry was not as evident, but significant asymmetry was observed throughout development in the medial and lateral orbitofrontal cortex and the insula as previously mentioned (Fig. 11). Age related asymmetries were observed in cortical gray matter volume development. Significant early age asymmetry was observed in the precuneus and the temporal pole, and late age asymmetry was observed in the superior temporal gyrus, entorhinal cortex, superior frontal cortex, and parahippocampal gyrus (Fig. 12).

### Sex differences in cortical development

**Fig. 13:**
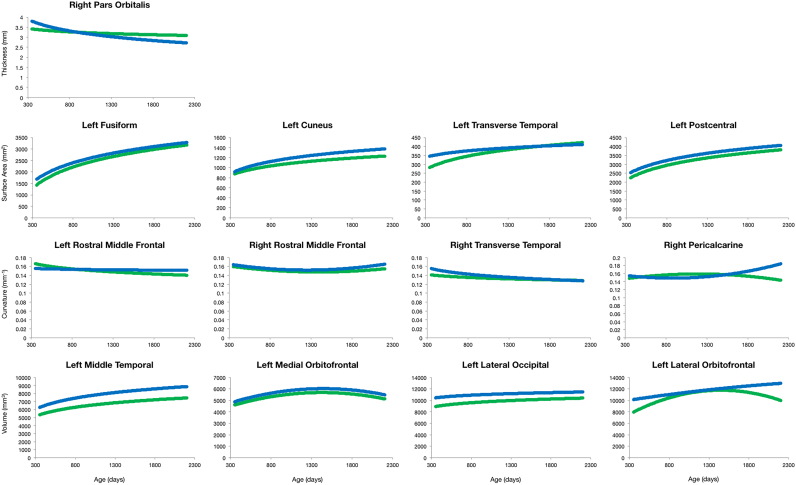
Differential cortical development based on sex. First row represents growth trajectories of cortical thickness; the second row represents growth trajectories of cortical surface area; the third row represents growth trajectories of cortical curvature; and the fourth represents growth trajectories of cortical gray matter volume. The blue line represents males and the green line represents the females.

Our cohort consisted of 62 females and 78 males. Representative scatter plots of differential cortical maturation curves divided by sex are illustrated in Fig. 13, while a full summary of the analyzed brain regions is provided in Supplementary Table 6. Differential developmental trajectories segregated by sex were generated for cortical thickness, surface area, mean curvature, and volume for all brain regions using the appropriate region specific model. For cortical thickness significant difference between male and female growth curves was observed in the right pars orbitalis. Widespread significant differences in surface area development based on sex were observed throughout the brain. Specific regions that showed bilateral differential surface area growth curves include the fusiform gyrus, cuneus, postcentral gyrus, transverse temporal gyrus, among others (Supplementary Table 6). With regards to curvature, significant developmental difference between sexes was observed in the left rostral middle frontal cortex, right transverse temporal, right pericalcarine, and right middle rostral frontal. Volume development also exhibited widespread significant differences based on sex. Some specific regions include the middle temporal gyrus bilaterally, the medial and lateral orbitofrontal cortex bilaterally, and the left lateral occipital, but like surface area extend beyond those listed here.

## Discussion

In this work we have sought to bridge the knowledge gap in cortical maturation that currently exists between infants and neonates (generally under 2 years of age) (Gilmore et al., 2012, Knickmeyer et al., 2008, Lyall et al., 2015) and later childhood/adolescence (generally 5–8 years of age and above) (Raznahan et al., 2011, Shaw et al., 2008, Sowell et al., 2004) by characterizing patterns of cortical growth from 1 to 6 years of age. Results of our analysis reveal that cortical thickness, surface area, curvature, and volume change with age throughout early childhood, exhibiting region specific developmental trajectories and asymmetries. These results provide new insight into typical early neurodevelopment, and may allow for future research on variable and abnormal cortical brain maturation.

Prior studies on cortical maturation in neonates have shown significant changes in cortical thickness over the first two years of development. The increase in cortical thickness from birth to one year of age is followed by region-specific thinning (bilateral anterior cingulate gyrus, bilateral middle cingulate gyrus, right gyrus rectus, and left rolandic operculum) from 1 to 2 years, at which point cortical thickness has reached 97.1% of adult values (Lyall et al., 2015, Nie et al., 2014). Our results reveal that this early cortical expansion shifts to a period of cortical thinning from 1–5 years of age, followed by thickening of posterior brain regions between 5 and 6 years of age. Prior analysis performed by our group highlights the concurrency of this cortical development pattern with logarithmic increases in the myelination of both cortical and adjacent white matter between ages 1 and 6, suggesting that cortical development and myelination are complementary, yet distinct, processes during this time (Croteau-Chonka et al., 2016). In children older than 6, region-specific linear and nonlinear cortical thickness development patterns remain (Shaw et al., 2008), with peak values observed during adolescence (Raznahan et al., 2011). Notably, association cortices reach peak thickness values later than sensory cortices (Brown et al., 2012, Raznahan et al., 2011, Sowell et al., 2004). The developmental pattern that precedes these peaks must include minimum values of cortical thickness. Our results show the time points at which sensory cortices reach their minimum values, however the time points at which association cortices reach their minimum values is still unknown. Longitudinal studies are needed to further characterize the nonlinear behavior of cortical thickness growth.

Neonates show rapid expansions from baseline cortical surface area of about 114.6%, reaching 69.1% of adult values by age 2 (Lyall et al., 2015). Whole brain surface area has been observed to follow a nonuniform 2 to 4 fold expansion from birth to adulthood (Hill et al., 2010a, Hill et al., 2010b) with peak surface area occurring around 8.1 years in females and 9.7 years in males (Raznahan et al., 2011). Our results expand upon this work and show an average 51% increase in cortical surface area from 1 to 6 years of age. Specifically, we show that these increases are nonlinear in nature, with region-specific logarithmic or quadratic trajectories. Such findings provide new insight into a critical period of cortical surface area maturation.

Changes in cortical folding have been observed in neonates through analysis of gyrification index (Li et al., 2014) and deep sulcal landmarks (Meng et al., 2014), highlighting a 16.1% increase in gyrification over the first year of life and a 6.1% increase in the second year. Li et al. also describe significant heterogeneity in neonatal cortical folding with the greatest changes observed in association cortices and the least changes in sensorimotor, visual, and auditory cortices. Our results further demonstrate that there is minimal change (<1%) in sensorimotor cortical curvature over the first 6 years of development. Our analysis of cortical curvature is preliminary and additional studies are needed to understand the early development trajectories of different cortical folding metrics such as gyrification and sulcation.

Prior research on cortical gray matter volume has revealed that cortical regions show distinct maturation trajectories over the first two years of life with a 108% increase in volume over the first year and a 19% increase in the second year (Gilmore et al., 2012). Our results confirm and expand upon these findings, showing that volume development between ages 1 and 6 follows distinct trajectories based on neuroanatomical location. Although we observed overall increases in gray matter volume across the whole brain, our results raise questions regarding differential rates of cortical volume expansion as well as within-subject variability in cortical volume development.

Prior studies have shown significant differences in cortical development trajectories between atypical pediatric populations and age-matched controls. One example is schizophrenia, a potentially heritable neurodevelopmental disorder that typically manifests in late adolescence or early adulthood. Neonates at high genetic risk for the disorder exhibit significant decreases in cortical thickness compared to controls (Li et al., 2016), a pattern that has also been observed in a population ages 7–26 diagnosed with childhood onset schizophrenia (Greenstein et al., 2006). These results highlight the utility of measures such as cortical thickness and surface area for investigating aberrant brain growth. Similarly to our investigations of typical development, measures of cortical morphometry in populations with genetic or environmental risk for disease pathology can contribute to the delineation between typical and atypical brain development.

Asymmetry in brain development has been observed over the first two years of life when measured through gray matter maturation (Gilmore et al., 2007), cortical thickness (Li et al., 2015), mean curvature, and surface area (Li et al., 2014). With regards to cortical thickness, leftward asymmetry is more prominent in the prefrontal, paracentral, and anterior cingulate cortices and increases in magnitude over the first two years of development; while, rightward asymmetry is evident in the lateral temporal and posterior insula and decreases in magnitude after birth (Li et al., 2015). Our results reveal that this leftward asymmetry, evident from birth to 2 years of age, persists in the anterior cingulate with additional leftward asymmetries appearing in the lateral orbitofrontal cortex and precentral gyrus over the first 6 years of development. Our cohort also reveals significant rightward asymmetry in cortical thickness development in the precuneus and lateral occipital cortex.

Leftward asymmetries in surface area development over the first two years of life have been identified in the superior frontal cortex, poster central sulcus, and temporal pole; while rightward asymmetries were localized to the superior temporal sulcus, inferior parietal cortex, cuneus, and parietoccipital sulcus (Li et al., 2015). We reveal that these significant neonatal leftward asymmetries remain constant between the ages of 1 and 6 with respect to the superior temporal sulcus and superior frontal gyrus. Additionally, we show significant leftward developmental asymmetries in the caudal middle frontal gyrus, entorhinal cortex, medial orbitofrontal cortex, and transverse temporal gyrus. Reasons for discrepancies between our results and those of prior studies on early cortical asymmetry are unclear. Longitudinal studies that encompass the entire period between birth and age 6 are needed to further characterize patterns of asymmetry and resolve these discrepancies.

Differences in cortical maturation, specifically cortical thickness, between sex has been observed in older cohorts over a large age range (8–87 years old) (Sowell et al., 2007); while, sex differences in cortical thickness were not apparent in cohorts of narrow age ranges (10–14 years old) (Bramen et al., 2012). In participants age 8 to 30 years of age sex differences have been observed in cortical surface area, with males showing greater surface area in frontal, parietal, temporal lobe, and anterior cingulate up to the age of 15 years (Koolschiin and Crone, 2013). With regards to cortical gray matter volume, Gilmore et al. (2007) demonstrated that sexual dimorphism is present at birth. Our findings extend these results and reveal that gray matter volume sexual dimorphisms continue throughout the brain from ages 1 to 6. Here, our results extend the findings of both Gilmore et al. and Bramen et al., providing greater insight on early brain maturation by demonstrating minimal sex differences in cortical thickness development; while, widespread significant sex differences are observed in cortical surface area, curvature, and volume development. Moreover we are the first study to classify region specific sex developmental differences in a cohort of participants ages 1 to 6 and reveal that temporal lobe, occipital lobe, and frontal lobe structures all exhibit differences in surface area, curvature, and volume development. Reasons for these trends are unclear but could range from altered effects of estrogen and testosterone on brain structure and synaptogenesis to variability in social emotional environments throughout early development (Balthazart and Ball, 2006, Milner et al., 2001). Future research is needed to consider these and additional factors influencing sex based cortical maturation.

Cytoarchitectural divisions of the cortex have been shown to correlate with developmental trajectories in adolescent populations (Shaw et al., 2008). While our results reveal anatomical associations between developmental trajectories, the Freesurfer segmentation protocol limits analysis of development in anatomy defined by cytoarchitecture i.e. allocortex, transition cortex, granular, and agranular neocortex. We hope for future studies to refine the anatomical segmentation protocol and correlate our results with cytoarchitectonic features of the cortex.

A potential concern in our study involves the use of Freesurfer (Fischl, 2012) for cortical reconstruction in children under 4 years old. Prior research from our group has successfully demonstrated using Freesurfer cortical reconstruction to generate a cortical ribbon from a single (IR-SPGR) contrast acquisition for analysis of cortical myelin and thickness development between ages 1 and 6 (Croteau-Chonka et al., 2016, Deoni et al., 2015). Success with Freesurfer has also been reported in toddlers at 12 months of age (Travis et al., 2014). Children under 1 year of age were excluded from this study to prevent inaccurate cortical segmentation due to lack of contrast between gray and white matter at this early stage of development. We also recognize that there is variability between cortical values obtained from Freesurfer with other diffeomorphic volume based algorithms such as ANTs (Tustison et al., 2014), but Freesurfer has been shown to provide a significantly better alignment of cortical landmarks than volume based algorithms in cohorts of younger participants age 4–11 years old (Ghosh et al., 2010). Another potential concern of our analysis was the use of MRI images for cortical segmentation that were greater than 1 mm^3^ isotropic resolution, the recommended resolution for adult studies. Yet, prior studies of pediatric neurodevelopment report successful analysis of cortical maturation using MR data acquired at voxel resolutions equal and greater than the resolution of the images analyzed in the current study (1.2×1.2×1.2 mm^3^) (Deoni et al., 2015, Shaw et al., 2008, Shaw et al., 2012), giving us confidence that such voxel resolution is suitable.

A final concern for our study was the use of a novel anatomic template that was designed based on the age range of our cohort to be used in the Freesurfer cortical reconstruction pipeline. We have used this template in prior studies of cortical myelin development (Croteau-Chonka et al., 2016; Deoni et al., 2015) and successfully obtained robust cortical segmentation. In all 3 studies the same 5 male and 5 female subjects from each 1–2 years, 2–3 years, 3–4 years, 4–5 years, and 5–6 years age groups were used to generate our novel study template, enabling feasibility of studying cortical development in our younger cohort (Croteau-Chonka et al., 2016; Deoni et al., 2015). We do recognize in developing this template that averages of MRI scans across differing age ranges that undergo dynamic brain change were computed. However, this strategy of template creation mirrors our prior work that has been shown to successfully create structural and quantitative MRI templates of the pediatric brain (Deoni et al., 2012, Dean et al., 2014).

As a foundation study for typical brain development, we characterized and classified the dynamic and complex changes in cortical maturation that occur in children between 1 to 6 years of age. As a result, we sought to observe overall population trajectories eliminating bias from individual trajectories, and only included single time point data. Future studies will include multiple time point data and use the trajectories described here as a guideline to consider individual variations and potential aberrancies in development Prior studies in children of older cohorts (Shaw et al., 2006) reveal differential cortical maturation when stratified by IQ scores. We believe that the results presented here in a cohort of children with average ELC, VDQ, and NVDQ scores (surrogate measures of overall IQ, verbal IQ, and non verbal IQ respectively) are an important step in providing a foundation for future studies to explore cognitive anatomical relationships in infants and young children.

Overall, we found that cortical maturation between the ages of 1 and 6 is a complex and dynamic process that occurs with changes in cortical thickness, surface area, curvature, and gray matter volume. We observed clear differences in cortical growth trajectories based on neuroanatomical location as well as region specific asymmetries in cortical development. Our observations on cortical maturation are the first to describe and characterize cortical change between the ages of 2 and 6 and provide insight on an important and vulnerable period of neurodevelopment.

## Appendix A Supplementary material

Supplementary material Supplementary Table 1: Overview of a subset of prior studies on anatomical cortical development. in associated age ranges.

.

Supplementary material Supplementary Table 2: Analysis of different functions to describe change of cortical thickness with respect to age based on lowest BIC value. Additional analysis of percent change in cortical thickness from 1 to 6 years of age.

.

Supplementary material Supplementary Table 3: Analysis of different functions to describe change of cortical surface area with respect to age based on lowest BIC value. Additional analysis of percent change in cortical thickness from 1 to 6 years of age.

.

Supplementary material Supplementary Table 4: Analysis of different functions to describe change of cortical mean curvature with respect to age based on lowest BIC value. Additional analysis of percent change in cortical thickness from 1 to 6 years of age.

.

Supplementary material Supplementary Table 5: Analysis of different functions to describe change of cortical gray matter volume with respect to age based on lowest BIC value. Additional analysis of percent change in cortical thickness from 1 to 6 years of age.

.

Supplementary material Supplementary Table 6: P-values of F-Tests for analysis of differential cortical development based on sex.
