# Supplementary material for: Quantifying cortical development in typically developing toddlers and young children, 1–6 years of age
Source: Neuroimage. 2017 Jun;153:246–61. doi: 10.1016/j.neuroimage.2017.04.010 (PMC5460988; doi:10.1016/j.neuroimage.2017.04.010)
Supplement: Supplementary file 2 — Supplementary material Supplementary Table 2: Analysis of different functions to describe change of cortical thickness with respect to age based on lowest BIC value. Additional analysis of percent change in cortical thickness from 1 to 6 years of age. [file mmc2.docx]

| Thickness |  |  |  |  |  |
| --- | --- | --- | --- | --- | --- |
| corticalRegion | th.logarithmicBIC | th.quadraticBIC | th.linearBIC | th.BestFit | % change |
| (left) caudalanteriorcingulate | 134.57 | 138.98 | 134.41 | linear | -12.61 |
| (left) frontalpole | 295.42 | 299.47 | 295.15 | linear | -5.91 |
| (left) insula | -21.47 | -19.01 | -23.8 | linear | -8.47 |
| (left) lateralorbitofrontal | 67.27 | 68.9 | 65.75 | linear | -6.45 |
| (left) parsorbitalis | 180.8 | 184.86 | 180 | linear | -22.53 |
| (left) posteriorcingulate | 18.1 | 19.25 | 15.94 | linear | -7.27 |
| (left) precentral | -62.79 | -58.53 | -63.36 | linear | -10.11 |
| (left) precuneus | 0.26 | 4.08 | -0.83 | linear | -10.64 |
| (left) superiorfrontal | 38.31 | 39.94 | 35 | linear | -17.36 |
| (left) superiortemporal | 25.77 | 30.34 | 25.4 | linear | -4.64 |
| (left) temporalpole | 257.66 | 262.54 | 257.61 | linear | 3.66 |
| (right) caudalmiddlefrontal | 72.11 | 75.88 | 71 | linear | -10.59 |
| (right) frontalpole | 296.87 | 300.41 | 296.08 | linear | -15.70 |
| (right) lateralorbitofrontal | 121.21 | 121.14 | 119.12 | linear | -8.91 |
| (right) parahippocampal | 139.15 | 142.25 | 139.1 | linear | -0.95 |
| (right) parsopercularis | 68.92 | 71.37 | 67.35 | linear | -9.49 |
| (right) postcentral | 19.59 | 24.25 | 19.48 | linear | -13.70 |
| (right) precentral | -49.47 | -45.84 | -50.61 | linear | -7.34 |
| (right) precuneus | -13.15 | -11.39 | -16.09 | linear | -10.37 |
| (right) superiorparietal | -15.13 | -10.51 | -15.25 | linear | -9.34 |
| (right) temporalpole | 271.54 | 272.85 | 270.72 | linear | 7.49 |
| (left) bankssts | 160.05 | 163.15 | 161.22 | logarithmic | -7.72 |
| (left) caudalmiddlefrontal | 41.11 | 46.23 | 44.34 | logarithmic | -18.78 |
| (left) entorhinal | 197.57 | 202.39 | 197.7 | logarithmic | -1.96 |
| (left) fusiform | -7.43 | -0.34 | -1.71 | logarithmic | -15.32 |
| (left) inferiorparietal | 48.79 | 52.37 | 50.02 | logarithmic | -10.43 |
| (left) inferiortemporal | 69.38 | 74.47 | 72.97 | logarithmic | -18.29 |
| (left) isthmuscingulate | 61.19 | 66.62 | 67.25 | logarithmic | -17.12 |
| (left) medialorbitofrontal | 106.86 | 111.89 | 111.08 | logarithmic | -17.07 |
| (left) middletemporal | 102.47 | 104.37 | 107.32 | logarithmic | -16.58 |
| (left) paracentral | 37.75 | 42.18 | 37.81 | logarithmic | -12.37 |
| (left) parahippocampal | 163.82 | 168.82 | 163.88 | logarithmic | -7.32 |
| (left) parsopercularis | 52.45 | 55.63 | 58.05 | logarithmic | -16.05 |
| (left) parstriangularis | 106.6 | 109.76 | 111 | logarithmic | -20.00 |
| (left) postcentral | -18.15 | -14.54 | -16.06 | logarithmic | -12.70 |
| (left) rostralanteriorcingulate | 45.82 | 49.3 | 46.19 | logarithmic | -23.04 |
| (left) rostralmiddlefrontal | 83.47 | 87.15 | 86.58 | logarithmic | -22.29 |
| (left) superiorparietal | -54.23 | -49.96 | -54.21 | logarithmic | -8.43 |
| (left) supramarginal | 53.1 | 55.59 | 57.61 | logarithmic | -14.32 |
| (left) transversetemporal | 96.66 | 101.75 | 97.39 | logarithmic | -9.63 |
| (right) bankssts | 183.2 | 186.37 | 186.94 | logarithmic | -20.75 |
| (right) caudalanteriorcingulate | 110.4 | 114.15 | 115.57 | logarithmic | -18.60 |
| (right) cuneus | 67.61 | 72.28 | 69.55 | logarithmic | -13.77 |
| (right) entorhinal | 181.78 | 185.99 | 182.03 | logarithmic | 5.76 |
| (right) fusiform | -15.45 | -13.66 | -1.94 | logarithmic | -17.06 |
| (right) inferiorparietal | 66.32 | 69.77 | 68.74 | logarithmic | -14.28 |
| (right) inferiortemporal | 59.94 | 64.79 | 68.08 | logarithmic | -20.79 |
| (right) insula | 29.46 | 34.29 | 29.51 | logarithmic | -8.87 |
| (right) isthmuscingulate | 67.35 | 70.52 | 75.56 | logarithmic | -16.49 |
| (right) medialorbitofrontal | 124.55 | 129.28 | 128.16 | logarithmic | -17.80 |
| (right) middletemporal | 99.23 | 104.31 | 105.05 | logarithmic | -21.50 |
| (right) paracentral | 48.38 | 52.69 | 48.72 | logarithmic | -9.33 |
| (right) parsorbitalis | 218.55 | 223.27 | 220.59 | logarithmic | -19.30 |
| (right) parstriangularis | 130.28 | 135.36 | 131.5 | logarithmic | -13.23 |
| (right) posteriorcingulate | 50.05 | 55.08 | 50.57 | logarithmic | -8.74 |
| (right) rostralanteriorcingulate | 84.97 | 86.99 | 99.4 | logarithmic | -23.56 |
| (right) rostralmiddlefrontal | 117.61 | 122.27 | 120.73 | logarithmic | -19.17 |
| (right) superiorfrontal | 48.12 | 52.24 | 48.64 | logarithmic | -17.70 |
| (right) superiortemporal | 14.74 | 19.73 | 15.63 | logarithmic | -11.48 |
| (right) supramarginal | 17.91 | 22.82 | 18.97 | logarithmic | -14.18 |
| (right) transversetemporal | 121.63 | 126.48 | 122.07 | logarithmic | -13.65 |
| (left) cuneus | 63.69 | 61.61 | 73.69 | quadratic | 79.26 |
| (left) lateraloccipital | -13.57 | -15.44 | -7.84 | quadratic | 51.77 |
| (left) lingual | 8.98 | 7.14 | 18.41 | quadratic | 60.86 |
| (left) pericalcarine | 24.58 | 20.49 | 29.73 | quadratic | 74.28 |
| (right) lateraloccipital | 12.82 | 10.75 | 18.44 | quadratic | 54.10 |
| (right) lingual | 36.82 | 32.5 | 47.42 | quadratic | 72.58 |
| (right) pericalcarine | 90.01 | 86.36 | 93.63 | quadratic | 88.61 |
