# Supplementary material for: Quantifying cortical development in typically developing toddlers and young children, 1–6 years of age
Source: Neuroimage. 2017 Jun;153:246–61. doi: 10.1016/j.neuroimage.2017.04.010 (PMC5460988; doi:10.1016/j.neuroimage.2017.04.010)
Supplement: Supplementary file 3 — Supplementary material Supplementary Table 3: Analysis of different functions to describe change of cortical surface area with respect to age based on lowest BIC value. Additional analysis of percent change in cortical thickness from 1 to 6 years of age. [file mmc3.docx]

| Surface Area |  |  |  |  |  |
| --- | --- | --- | --- | --- | --- |
| corticalRegion | sa.logarithmicBIC | sa.quadraticBIC | sa.linearBIC | sa.BestFit | % Change |
| (left) bankssts | 1833.24 | 1837.41 | 1837.63 | logarithmic | 69.09 |
| (left) caudalanteriorcingulate | 1650.48 | 1657.56 | 1657.89 | logarithmic | 67.80 |
| (left) caudalmiddlefrontal | 2020.28 | 2023.82 | 2026.85 | logarithmic | 59.16 |
| (left) cuneus | 1910.87 | 1911.86 | 1918.22 | logarithmic | 44.85 |
| (left) frontalpole | 1390.26 | 1394.07 | 1394.21 | logarithmic | 37.25 |
| (left) fusiform | 2100.82 | 2107.01 | 2112.23 | logarithmic | 102.96 |
| (left) inferiorparietal | 2223.59 | 2227.26 | 2229.72 | logarithmic | 69.82 |
| (left) inferiortemporal | 2090.14 | 2092.68 | 2097.39 | logarithmic | 72.05 |
| (left) insula | 1977.53 | 1983.4 | 1979.85 | logarithmic | 31.62 |
| (left) lateralorbitofrontal | 2066.55 | 2067.27 | 2071.7 | logarithmic | 28.79 |
| (left) lingual | 2146.24 | 2146.55 | 2159.53 | logarithmic | 87.48 |
| (left) middletemporal | 2047.19 | 2049.89 | 2055.86 | logarithmic | 76.33 |
| (left) paracentral | 1892.82 | 1896 | 1898.29 | logarithmic | 47.65 |
| (left) parahippocampal | 1794.17 | 1796.83 | 1801.03 | logarithmic | 74.59 |
| (left) parsopercularis | 1920.46 | 1923.65 | 1928.47 | logarithmic | 53.74 |
| (left) parsorbitalis | 1716.12 | 1717.02 | 1721.3 | logarithmic | 40.56 |
| (left) parstriangularis | 1831.86 | 1837.9 | 1837.75 | logarithmic | 70.13 |
| (left) postcentral | 2177.84 | 2181.97 | 2182.28 | logarithmic | 63.81 |
| (left) posteriorcingulate | 1801.86 | 1807.38 | 1813.97 | logarithmic | 56.07 |
| (left) precentral | 2167.66 | 2172.19 | 2171.94 | logarithmic | 55.30 |
| (left) precuneus | 2137.09 | 2138.54 | 2152.98 | logarithmic | 66.31 |
| (left) rostralanteriorcingulate | 1762.48 | 1763.58 | 1776.1 | logarithmic | 108.08 |
| (left) rostralmiddlefrontal | 2253.02 | 2253.85 | 2263.96 | logarithmic | 55.34 |
| (left) superiorparietal | 2265.61 | 2269.88 | 2271.79 | logarithmic | 46.74 |
| (left) superiortemporal | 2126.26 | 2130.72 | 2130.66 | logarithmic | 52.53 |
| (left) supramarginal | 2132.9 | 2137.27 | 2139.52 | logarithmic | 59.93 |
| (left) temporalpole | 1628.03 | 1633.18 | 1628.7 | logarithmic | 18.39 |
| (left) transversetemporal | 1590.31 | 1595.57 | 1592.76 | logarithmic | 31.96 |
| (right) bankssts | 1791.92 | 1796.99 | 1793.02 | logarithmic | 46.73 |
| (right) caudalanteriorcingulate | 1908.48 | 1913.07 | 1910.48 | logarithmic | 77.35 |
| (right) caudalmiddlefrontal | 2004.56 | 2008.37 | 2007.49 | logarithmic | 46.47 |
| (right) cuneus | 1918.62 | 1918.65 | 1926.48 | logarithmic | 41.64 |
| (right) entorhinal | 1698.17 | 1699.87 | 1702.39 | logarithmic | 51.23 |
| (right) frontalpole | 1463.53 | 1467.93 | 1464.2 | logarithmic | 28.89 |
| (right) fusiform | 2141.75 | 2144.4 | 2151.99 | logarithmic | 83.55 |
| (right) inferiorparietal | 2253.81 | 2254.38 | 2262.49 | logarithmic | 54.31 |
| (right) inferiortemporal | 2126.51 | 2128.31 | 2136.66 | logarithmic | 88.98 |
| (right) insula | 2060.51 | 2062.4 | 2065.29 | logarithmic | 37.64 |
| (right) lateraloccipital | 2196.29 | 2198.48 | 2205.86 | logarithmic | 54.83 |
| (right) lateralorbitofrontal | 2105.8 | 2106.75 | 2111.75 | logarithmic | 38.46 |
| (right) middletemporal | 2093.3 | 2095.55 | 2103.81 | logarithmic | 74.99 |
| (right) paracentral | 1888.88 | 1890.15 | 1897.99 | logarithmic | 45.39 |
| (right) parahippocampal | 1802.79 | 1807.15 | 1807.87 | logarithmic | 61.60 |
| (right) parstriangularis | 1914.9 | 1916.15 | 1921.82 | logarithmic | 46.65 |
| (right) postcentral | 2163.26 | 2166.38 | 2171.71 | logarithmic | 59.31 |
| (right) precuneus | 2129.94 | 2133.52 | 2140.88 | logarithmic | 53.78 |
| (right) superiorparietal | 2232.48 | 2235.99 | 2238.87 | logarithmic | 43.74 |
| (right) superiortemporal | 2090.36 | 2091.07 | 2097.94 | logarithmic | 36.65 |
| (right) supramarginal | 2143.23 | 2147.58 | 2147.92 | logarithmic | 56.89 |
| (right) temporalpole | 1644.52 | 1646.45 | 1649.69 | logarithmic | 38.54 |
| (right) transversetemporal | 1566.05 | 1566.66 | 1569.19 | logarithmic | 29.71 |
| (left) entorhinal | 1717.35 | 1709.76 | 1725.35 | quadratic | 33.79 |
| (left) isthmuscingulate | 1829.26 | 1825.48 | 1837.87 | quadratic | 27.28 |
| (left) lateraloccipital | 2211.43 | 2210.36 | 2224.27 | quadratic | 41.24 |
| (left) medialorbitofrontal | 1957.51 | 1955.59 | 1970.19 | quadratic | 33.05 |
| (left) pericalcarine | 1965.08 | 1959.88 | 1974.36 | quadratic | 29.24 |
| (left) superiorfrontal | 2258.93 | 2254.44 | 2277.42 | quadratic | 46.91 |
| (right) isthmuscingulate | 1824.05 | 1819.99 | 1833.44 | quadratic | 29.70 |
| (right) lingual | 2138.14 | 2135.27 | 2150.8 | quadratic | 44.50 |
| (right) medialorbitofrontal | 1962.99 | 1956.38 | 1973.43 | quadratic | 24.61 |
| (right) parsopercularis | 1870.58 | 1870.43 | 1878.91 | quadratic | 33.14 |
| (right) parsorbitalis | 1745.29 | 1735.51 | 1756.02 | quadratic | 28.83 |
| (right) pericalcarine | 1990.55 | 1987.7 | 1996.93 | quadratic | 17.42 |
| (right) posteriorcingulate | 1908.24 | 1906.38 | 1919.37 | quadratic | 52.80 |
| (right) precentral | 2166.73 | 2163.78 | 2178.46 | quadratic | 37.92 |
| (right) rostralanteriorcingulate | 1812 | 1811.34 | 1822.27 | quadratic | 79.08 |
| (right) rostralmiddlefrontal | 2242.53 | 2238.67 | 2256.11 | quadratic | 40.25 |
| (right) superiorfrontal | 2265.16 | 2264.16 | 2278.91 | quadratic | 44.45 |
