# Supplementary material for: Quantifying cortical development in typically developing toddlers and young children, 1–6 years of age
Source: Neuroimage. 2017 Jun;153:246–61. doi: 10.1016/j.neuroimage.2017.04.010 (PMC5460988; doi:10.1016/j.neuroimage.2017.04.010)
Supplement: Supplementary file 4 — Supplementary material Supplementary Table 4: Analysis of different functions to describe change of cortical mean curvature with respect to age based on lowest BIC value. Additional analysis of percent change in cortical thickness from 1 to 6 years of age. [file mmc4.docx]

| Mean Curvature | |  |  |  |  |
| --- | --- | --- | --- | --- | --- |
| corticalRegion | mc.logarithmicBIC | mc.quadraticBIC | mc.linearBIC | mc.BestFit | %change |
| (left) caudalanteriorcingulate | -786.36 | -781.62 | -786.36 | linear | 1.50 |
| (left) insula | -731.1 | -728.08 | -732.76 | linear | 19.12 |
| (left) pericalcarine | -638.29 | -633.63 | -638.45 | linear | 6.49 |
| (right) caudalanteriorcingulate | -835.51 | -832.68 | -835.95 | linear | 2.27 |
| (right) insula | -697.83 | -695.75 | -700.11 | linear | 18.11 |
| (right) isthmuscingulate | -828.1 | -823.92 | -828.2 | linear | -1.12 |
| (right) medialorbitofrontal | -805.55 | -801.23 | -805.6 | linear | 0.95 |
| (right) parsorbitalis | -777.62 | -774.01 | -777.83 | linear | 1.45 |
| (right) precuneus | -910.08 | -909.7 | -910.8 | linear | 2.01 |
| (left) bankssts | -801.14 | -796.42 | -800.45 | logarithmic | 10.30 |
| (left) caudalmiddlefrontal | -836.18 | -835.83 | -834.23 | logarithmic | -5.73 |
| (left) cuneus | -725.34 | -723.02 | -724.78 | logarithmic | -2.83 |
| (left) entorhinal | -551.78 | -547.07 | -550.69 | logarithmic | -13.83 |
| (left) frontalpole | -618.64 | -613.54 | -618.19 | logarithmic | -4.07 |
| (left) fusiform | -753.46 | -752 | -750.86 | logarithmic | -8.45 |
| (left) inferiorparietal | -800.88 | -798.57 | -798.01 | logarithmic | -7.32 |
| (left) inferiortemporal | -813.33 | -812.16 | -811.31 | logarithmic | -6.02 |
| (left) isthmuscingulate | -792.64 | -788.04 | -791.85 | logarithmic | 3.91 |
| (left) lateraloccipital | -808.86 | -806.77 | -806.54 | logarithmic | -7.67 |
| (left) lateralorbitofrontal | -771.55 | -768.95 | -769.26 | logarithmic | -8.15 |
| (left) medialorbitofrontal | -759.06 | -757.02 | -758.88 | logarithmic | -1.19 |
| (left) middletemporal | -843.83 | -839.04 | -843.27 | logarithmic | -4.86 |
| (left) parahippocampal | -459.35 | -456.33 | -458.48 | logarithmic | -11.81 |
| (left) parsopercularis | -858.32 | -853.82 | -858.12 | logarithmic | -2.89 |
| (left) parsorbitalis | -707.07 | -701.83 | -705.33 | logarithmic | -8.48 |
| (left) parstriangularis | -835.5 | -832.88 | -835.04 | logarithmic | -1.78 |
| (left) posteriorcingulate | -839.52 | -834.98 | -839.08 | logarithmic | 4.21 |
| (left) rostralmiddlefrontal | -828.83 | -827.85 | -824.25 | logarithmic | -8.95 |
| (left) superiorfrontal | -888.04 | -885.34 | -886.8 | logarithmic | -3.43 |
| (left) superiortemporal | -836.1 | -835.78 | -833.1 | logarithmic | -7.57 |
| (left) supramarginal | -866.93 | -861.99 | -866.92 | logarithmic | -0.45 |
| (left) temporalpole | -565.44 | -565.08 | -562.79 | logarithmic | -15.80 |
| (left) transversetemporal | -747.65 | -742.93 | -747.44 | logarithmic | -7.45 |
| (right) bankssts | -741.65 | -739.99 | -741.6 | logarithmic | -1.14 |
| (right) cuneus | -705 | -702.54 | -703.83 | logarithmic | -5.08 |
| (right) entorhinal | -525.2 | -520.37 | -523.11 | logarithmic | -15.16 |
| (right) frontalpole | -632.06 | -630.26 | -631.47 | logarithmic | -4.20 |
| (right) fusiform | -821.01 | -818.35 | -817.23 | logarithmic | -8.25 |
| (right) inferiorparietal | -783.52 | -781.47 | -780.54 | logarithmic | -7.44 |
| (right) inferiortemporal | -816.62 | -814.48 | -813.03 | logarithmic | -7.66 |
| (right) lateraloccipital | -788.01 | -786.65 | -786.24 | logarithmic | -5.36 |
| (right) lateralorbitofrontal | -745.34 | -742.59 | -743.68 | logarithmic | -6.89 |
| (right) middletemporal | -845.15 | -841.66 | -843.32 | logarithmic | -6.90 |
| (right) parsopercularis | -861.3 | -858.64 | -860.88 | logarithmic | -2.18 |
| (right) parstriangularis | -828.01 | -825.22 | -827.53 | logarithmic | -2.32 |
| (right) postcentral | -837.87 | -837.07 | -836.45 | logarithmic | -4.24 |
| (right) rostralanteriorcingulate | -659.4 | -657.89 | -646.86 | logarithmic | 60.96 |
| (right) superiorfrontal | -922.76 | -919.08 | -921.81 | logarithmic | -2.70 |
| (right) superiortemporal | -863.44 | -863.2 | -858.52 | logarithmic | -9.39 |
| (right) supramarginal | -857.65 | -854 | -856.17 | logarithmic | -4.60 |
| (right) transversetemporal | -711.97 | -706.83 | -710.95 | logarithmic | -12.87 |
| (left) lingual | -765.57 | -768.21 | -763.33 | quadratic | -1.14 |
| (left) paracentral | -806.46 | -809.06 | -804.25 | quadratic | 0.01 |
| (left) postcentral | -842.46 | -843.3 | -841.04 | quadratic | 0.04 |
| (left) precentral | -900.86 | -904.64 | -898.11 | quadratic | -0.25 |
| (left) precuneus | -869.91 | -870.88 | -869.83 | quadratic | 2.69 |
| (left) rostralanteriorcingulate | -668.13 | -682.74 | -653.75 | quadratic | 21.66 |
| (left) superiorparietal | -829.82 | -835.43 | -826.64 | quadratic | -0.42 |
| (right) caudalmiddlefrontal | -860.2 | -862.35 | -856.71 | quadratic | -2.06 |
| (right) lingual | -708.98 | -715.93 | -708.11 | quadratic | 3.59 |
| (right) paracentral | -821.18 | -827.37 | -820.08 | quadratic | 2.96 |
| (right) parahippocampal | -546.44 | -550.17 | -542.99 | quadratic | -3.74 |
| (right) pericalcarine | -648.54 | -652.1 | -651.23 | quadratic | 15.12 |
| (right) posteriorcingulate | -860.42 | -864.56 | -864.27 | quadratic | 9.96 |
| (right) precentral | -906.88 | -917.44 | -903.57 | quadratic | 0.43 |
| (right) rostralmiddlefrontal | -845.31 | -845.47 | -842.98 | quadratic | -1.64 |
| (right) superiorparietal | -824.49 | -834.66 | -821.66 | quadratic | 1.68 |
| (right) temporalpole | -515.9 | -517.07 | -514.23 | quadratic | 0.18 |
