# Supplementary material for: Quantifying cortical development in typically developing toddlers and young children, 1–6 years of age
Source: Neuroimage. 2017 Jun;153:246–61. doi: 10.1016/j.neuroimage.2017.04.010 (PMC5460988; doi:10.1016/j.neuroimage.2017.04.010)
Supplement: Supplementary file 5 — Supplementary material Supplementary Table 5: Analysis of different functions to describe change of cortical gray matter volume with respect to age based on lowest BIC value. Additional analysis of percent change in cortical thickness from 1 to 6 years of age. [file mmc5.docx]

| Gray Matter Volume | |  |  |  |  |
| --- | --- | --- | --- | --- | --- |
| corticalRegion | vl.logarithmicBIC | vl.quadraticBIC | vl.linearBIC | vl.BestFit | %Change |
| (left) temporalpole | 2175.97 | 2180.8 | 2175.86 | linear | 9.66 |
| (right) bankssts | 2155.54 | 2160.13 | 2155.25 | linear | 15.21 |
| (right) lateraloccipital | 2507.22 | 2511.13 | 2506.26 | linear | 14.15 |
| (right) temporalpole | 2171.72 | 2174.16 | 2169.98 | linear | 33.06 |
| (left) bankssts | 2175.53 | 2180.15 | 2176.08 | logarithmic | 46.85 |
| (left) caudalanteriorcingulate | 2080.85 | 2085.55 | 2086.65 | logarithmic | 42.75 |
| (left) caudalmiddlefrontal | 2392.73 | 2396.43 | 2394.18 | logarithmic | 17.19 |
| (left) cuneus | 2171.07 | 2175.75 | 2171.13 | logarithmic | -1.38 |
| (left) frontalpole | 2030.74 | 2034.97 | 2031.29 | logarithmic | 28.78 |
| (left) fusiform | 2436.46 | 2440.49 | 2440.69 | logarithmic | 44.40 |
| (left) inferiorparietal | 2578.29 | 2582.44 | 2582.23 | logarithmic | 33.89 |
| (left) inferiortemporal | 2479.12 | 2482.77 | 2481.09 | logarithmic | 26.87 |
| (left) insula | 2283.72 | 2288.48 | 2286.43 | logarithmic | 15.21 |
| (left) isthmuscingulate | 2149.39 | 2151.43 | 2149.48 | logarithmic | 1.50 |
| (left) lateraloccipital | 2467.72 | 2472.35 | 2468.57 | logarithmic | 12.99 |
| (left) lingual | 2395.14 | 2399.87 | 2396.33 | logarithmic | 15.39 |
| (left) middletemporal | 2520.86 | 2525.53 | 2521.21 | logarithmic | 41.53 |
| (left) paracentral | 2195.99 | 2200.5 | 2197.08 | logarithmic | 16.11 |
| (left) parahippocampal | 2116.02 | 2118.43 | 2118.9 | logarithmic | 39.69 |
| (left) parsopercularis | 2314.01 | 2317.95 | 2315.44 | logarithmic | 16.04 |
| (left) parstriangularis | 2258.06 | 2263.38 | 2258.76 | logarithmic | 25.88 |
| (left) pericalcarine | 2102.45 | 2104.11 | 2103.55 | logarithmic | 8.92 |
| (left) postcentral | 2462.38 | 2467.44 | 2463.66 | logarithmic | 27.89 |
| (left) posteriorcingulate | 2167.81 | 2168.74 | 2174.92 | logarithmic | 29.80 |
| (left) precentral | 2463.45 | 2467.77 | 2466.18 | logarithmic | 25.35 |
| (left) superiorparietal | 2546.86 | 2551.02 | 2549.34 | logarithmic | 17.74 |
| (left) superiortemporal | 2513.37 | 2518.45 | 2515.63 | logarithmic | 33.06 |
| (left) supramarginal | 2532.75 | 2537.78 | 2533.48 | logarithmic | 19.99 |
| (left) transversetemporal | 1946.25 | 1951.35 | 1946.6 | logarithmic | 15.36 |
| (right) caudalanteriorcingulate | 2252.81 | 2257.86 | 2253.81 | logarithmic | 41.21 |
| (right) caudalmiddlefrontal | 2383.02 | 2386.73 | 2384.28 | logarithmic | 18.90 |
| (right) cuneus | 2204.82 | 2208.29 | 2205.13 | logarithmic | 4.25 |
| (right) entorhinal | 2062.45 | 2066.8 | 2063.71 | logarithmic | 50.52 |
| (right) frontalpole | 2095.13 | 2099.33 | 2095.13 | logarithmic | 0.52 |
| (right) fusiform | 2471.74 | 2476.15 | 2472.9 | logarithmic | 29.91 |
| (right) inferiorparietal | 2604.79 | 2608.33 | 2607.12 | logarithmic | 20.74 |
| (right) inferiortemporal | 2489.25 | 2493.12 | 2491.86 | logarithmic | 33.82 |
| (right) insula | 2344.93 | 2347.46 | 2347.8 | logarithmic | 18.82 |
| (right) isthmuscingulate | 2172.87 | 2174.18 | 2173.77 | logarithmic | 8.87 |
| (right) lingual | 2403.28 | 2407.48 | 2403.5 | logarithmic | 3.61 |
| (right) middletemporal | 2534.48 | 2538.82 | 2535.75 | logarithmic | 28.99 |
| (right) paracentral | 2244.64 | 2249.1 | 2246.17 | logarithmic | 16.32 |
| (right) parahippocampal | 2165.46 | 2168.39 | 2168.13 | logarithmic | 37.30 |
| (right) parsorbitalis | 2218.54 | 2219.63 | 2219.53 | logarithmic | 10.09 |
| (right) parstriangularis | 2307.63 | 2310.73 | 2309.35 | logarithmic | 18.65 |
| (right) pericalcarine | 2158.31 | 2163.02 | 2158.39 | logarithmic | 2.11 |
| (right) postcentral | 2452.02 | 2454.29 | 2455.87 | logarithmic | 25.53 |
| (right) precuneus | 2442.24 | 2443.74 | 2446.73 | logarithmic | 18.25 |
| (right) rostralanteriorcingulate | 2173.14 | 2174.28 | 2180.04 | logarithmic | 60.64 |
| (right) superiorparietal | 2559.58 | 2563.83 | 2560.91 | logarithmic | 16.10 |
| (right) superiortemporal | 2483.99 | 2486.78 | 2485.6 | logarithmic | 14.00 |
| (right) supramarginal | 2506.35 | 2510.45 | 2508.44 | logarithmic | 23.90 |
| (right) transversetemporal | 1888.82 | 1889.82 | 1889.93 | logarithmic | 11.18 |
| (left) entorhinal | 2068.7 | 2064.54 | 2074.3 | quadratic | 26.42 |
| (left) lateralorbitofrontal | 2373.61 | 2365.54 | 2380.3 | quadratic | 9.70 |
| (left) medialorbitofrontal | 2260.31 | 2257.27 | 2267.35 | quadratic | 11.18 |
| (left) parsorbitalis | 2159.64 | 2156.33 | 2160.89 | quadratic | -4.21 |
| (left) precuneus | 2462.61 | 2462.11 | 2468.65 | quadratic | 14.46 |
| (left) rostralanteriorcingulate | 2173.07 | 2167.63 | 2185 | quadratic | 40.95 |
| (left) rostralmiddlefrontal | 2637.48 | 2637.4 | 2641.06 | quadratic | 7.90 |
| (left) superiorfrontal | 2643.39 | 2635 | 2651.73 | quadratic | 11.16 |
| (right) lateralorbitofrontal | 2378.98 | 2366.1 | 2386.95 | quadratic | 8.58 |
| (right) medialorbitofrontal | 2274.86 | 2268.46 | 2278.39 | quadratic | 1.96 |
| (right) parsopercularis | 2232.18 | 2228.76 | 2236.97 | quadratic | 9.45 |
| (right) posteriorcingulate | 2229.59 | 2225.25 | 2238.15 | quadratic | 27.35 |
| (right) precentral | 2464.58 | 2462.37 | 2470.01 | quadratic | 14.10 |
| (right) rostralmiddlefrontal | 2632.33 | 2630.9 | 2637.25 | quadratic | 13.73 |
| (right) superiorfrontal | 2647.06 | 2646.28 | 2651.33 | quadratic | 8.92 |
